# *FLT3*-ITD allelic ratio and *HLF* expression predict FLT3 inhibitor efficacy in adult AML

**DOI:** 10.1038/s41598-021-03010-7

**Published:** 2021-12-07

**Authors:** Jarno Kivioja, Disha Malani, Ashwini Kumar, Mika Kontro, Alun Parsons, Olli Kallioniemi, Caroline A. Heckman

**Affiliations:** 1 Institute for Molecular Medicine Finland - FIMM, HiLIFE - Helsinki Institute of Life Science, iCAN Digital Precision Cancer Medicine Flagship, University of Helsinki, Tukholmankatu 8, P.O. Box 20, 00290 Helsinki, Finland; 2 Hematology Research Unit Helsinki, University of Helsinki, Helsinki, Finland; 3 Department of Hematology, Helsinki University Hospital Comprehensive Cancer Center, Helsinki, Finland; 4 Science for Life Laboratory, Department of Oncology and Pathology, Karolinska Institutet, Stockholm, Sweden

**Keywords:** Cancer, Drug discovery, Molecular biology, Molecular medicine

## Abstract

*FLT*3 internal tandem duplication (*FLT3*-ITD) is a frequent mutation in acute myeloid leukemia (AML) and remains a strong prognostic factor due to high rate of disease recurrence. Several *FLT3*-targeted agents have been developed, but determinants of variable responses to these agents remain understudied. Here, we investigated the role *FLT3*-ITD allelic ratio (ITD-AR), ITD length, and associated gene expression signatures on FLT3 inhibitor response in adult AML. We performed fragment analysis, ex vivo drug testing, and next generation sequencing (RNA, exome) to 119 samples from 87 AML patients and 13 healthy bone marrow controls. We found that ex vivo response to FLT3 inhibitors is significantly associated with ITD-AR, but not with ITD length. Interestingly, we found that the *HLF* gene is overexpressed in *FLT3*-ITD^+^ AML and associated with ITD-AR. The retrospective analysis of AML patients treated with FLT3 inhibitor sorafenib showed that patients with high *HLF* expression and ITD-AR had better clinical response to therapy compared to those with low ITD-AR and *HLF* expression. Thus, our findings suggest that *FLT3* ITD-AR together with increased *HLF* expression play a role in variable FLT3 inhibitor responses observed in *FLT3*-ITD^+^ AML patients.

## Introduction

Acute myeloid leukemia (AML) with Fms-like tyrosine kinase 3 internal tandem duplication (*FLT3*-ITD) represents a large and heterogeneous group of patients associated with unfavorable prognosis^1–4^. The recently approved type 1 FLT3 inhibitors have led to improved survival of *FLT3*-ITD^+^ AML patients over standard chemotherapy, however, initial responses are often variable and long-term benefits with either monotherapy or combination therapy are rarely achieved^5–8^. Hence, further studies are needed for identifying molecular biomarkers associated with variable FLT3 inhibitor responses. The aim of this study was to assess whether (i) *FLT3*-ITD allelic ratio (AR) and ITD mutation length and/or (ii) transcriptomic features, are associated with ex vivo and clinical responses to FLT3 inhibitors in adult AML. To address this topic and build upon our previously published work on *FLT3*-ITD^+^ AML patients carrying a co-operative *NUP98-NSD1* gene fusion^9^, we performed a systematic analysis by integrating ex vivo drug sensitivity and resistance testing (DSRT) with *FLT3* mutation fragment analysis, and next-generation sequencing (exome and RNA). These analyses were carried out using 119 samples from 87 adult AML patients (Table 1) and 13 healthy donors as specified in the Supplementary file.

**Table 1:** Patient characteristics.

	Total	*FLT3*-WT	*FLT3*-ITD
Total, no. (%)	87	49 (56)	38 (44)
**Type AML, no. (%)**			
De novo	67	37 (55)	30 (45)
Secondary	20	12 (60)	8 (40)
Median age at dg (years)	62.7	64.2	58.7
**Gender, no. (%)**			
Male	40	22 (55)	18 (45)
Female	47	27 (57)	20 (43)
Samples, no. (%)	119	68 (57)	51 (43)
Diagnostic	57	35 (61)	22 (39)
Rel./Ref	62	33 (53)	29 (47)
Median BM blast (%)	60	57.5	60

We initially characterized ITD-AR (mutant/total *FLT3*) and ITD lengths from 119 gDNA samples collected from 38 *FLT3*-ITD^+^ patients and 49 *FLT3*-ITD^-^ controls. The analysis identified ITD-AR and ITD lengths from all 38 *FLT3*-ITD^+^ patients including 22 samples collected at diagnosis (43%) and 29 at relapsed/refractory (R/R) stage (57%). Altogether 29 unique gain-of-function ITD mutations were found ranging from 17 to 213 bp in length (median 45 bp) with the most frequent size being 21 bp. Five patients (13%) carried two distinct ITD mutations and one had bi-allelic ITD (ITD-AR 1.0). In seven patients ITD mutations either appeared or disappeared during disease progression indicating changes in clonal dominance. In the *FLT3*-ITD^+^ cohort, ITD-AR varied between 0.016 and 1.0 (median, 0.338; 95% CI 0.27–0.41) (Supplemental Table 1). Interestingly, 36% of the diagnostic samples (8/22) had high ITD-AR (> 0.338). We found no correlation between ITD-AR and ITD length, however, weak positive correlation existed between ITD-AR and BM blast percentage (Fig. S1). Both ITD-AR and ITD length showed similar frequencies across males and females as well as between AML patients below or above 65 years of age, respectively (Fig. S2). The R/R AML patients (n = 29) had significantly higher ITD-AR (*P* = 0.024; median: 0.39; 95% CI 0.302–0.478) compared to unmatched diagnostic samples (n = 22) (median: 0.27; 95% CI 0.164–0.369), which indicates that cellular addiction on activated FLT3-signaling increases during disease progression.

We next assessed the impact of ITD-AR and ITD length on leukocyte and BM blast counts, which are frequently increased in *FLT3*-ITD^+^ AML^10^. In samples divided based on median ITD-AR, the ITD-AR^high^ (> 0.338) group had significantly higher blast counts compared to the ITD-AR^low^ (< 0.338) group (Fig. S2). The ITD length on the contrary impacted both variables. Blast counts were significantly higher in the ITD^long^ (≥ 45 bp) group compared to the ITD^short^ (< 45 bp) group (*P* = 0.0431), while the ITD^long^ patients also showed elevated blood leukocyte counts (*P* = 0.0284) compared to ITD^short^ patients (< 45 bp). No significant difference in overall survival (OS) existed between de novo* FLT3*-ITD^+^ patients with either long or short ITD. The survival analysis included both diagnostic and R/R samples, considering that ITD lengths remain unchanged for the majority of patients with *FLT3*-ITD persistence at disease progression^8^. Importantly, we found significant difference in OS (*P* = 0.024) between de novo AML patients having either low or high ITD-AR at diagnosis. The 3-year OS rate was only 17% in ITD-AR^high^ compared to 67% in the ITD-AR^low^ group (Fig. S3). In conclusion, these analyses provide evidence that R/R *FLT3*-ITD^+^ samples frequently have higher ITD-AR and blast counts compared to diagnostic samples, whereas patients with long ITD mutations have higher leukocyte counts compared to patients with short ITD (< 45 bp) regardless of disease stage.

**Figure 1:**
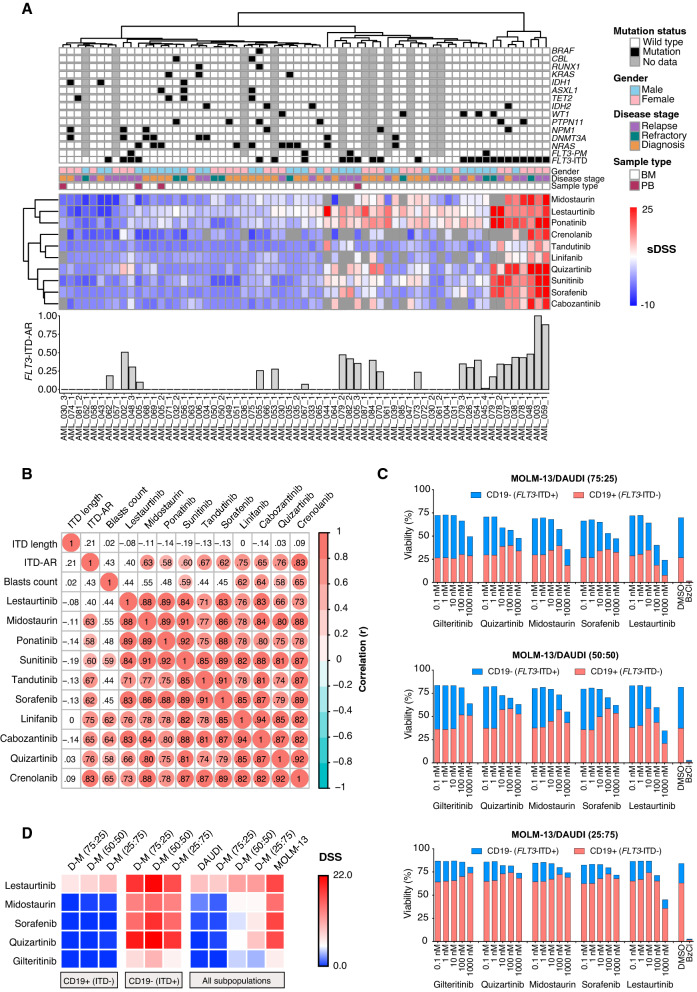
*FLT3*-ITD allelic ratio impacts FLT3 inhibitor responses in AML. (**A**) The top panel shows the presence (black) or absence (white) of recurrent somatic mutations in AML detected by exome sequencing. The *FLT3*-ITD mutations were detected using fragment analysis. The middle panel shows gender, disease stage, and sample type for the tested samples. The heatmap shows clustering of FLT3 inhibitor responses (sDSS) in AML samples with (N = 25) and without (N = 40) *FLT3*-ITD compared to healthy controls (N = 13). Blue color indicates resistance and red indicates sensitivity compared to healthy controls. The hierarchical clustering of samples and sDSS was performed using Euclidean distance matrix and complete clustering method. The bar plot below the heatmap shows *FLT3*-ITD-AR (mutant/total *FLT3*) in the *FLT3*-ITD^+^ samples. (**B**) The matrix shows correlation between FLT3 inhibitor response, ITD-AR, ITD length, and blast count in 25 *FLT3*-ITD^+^ AML samples. The analysis was performed by Pearson correlation. Red circles indicate significant (*P* < 0.05) correlation with the depth of the color referring to the correlation coefficients. The results show that ITD-AR has the highest correlation with the most specific FLT3 inhibitors (quizartinib and crenolanib), whereas ITD length lacks correlation with FLT3 inhibitor response. (**C**) Stacked bar-plot representation of viable (Annexin V-) cell counts after 72 h treatment of three different mixtures of *FLT3*-ITD^+^ (MOLM-13) and *FLT3*-ITD^-^ (DAUDI) cell lines with five FLT3 inhibitors as measured by flow cytometry. Each bar shows a percentage of live CD19- (MOLM-13) and CD19 + (DAUDI) cells compared to total number of acquired singlet cells (100%). The percentages were calculated from two repeated measurements. (**D**) The heatmap displays DSS of five FLT3 inhibitors in CD19 + (*FLT3*-ITD^-^), CD19- (*FLT3*-ITD^+^), and all viable cells of DAUDI and MOLM-13 cell lines or their co-cultures after the 72 h drug treatment. The DSS was calculated for the inhibitors from modified area under a five-point dose–response curve using % inhibition values at each concentration as described in the online Supplementary file. All experiments were performed in duplicates.

After the initial characterization, we performed ex vivo drug response analysis of BM mononuclear cells from 65 patients (25 *FLT3*-ITD^+^ and 40 *FLT3*-ITD^-^) using a DSRT-assay^9,11^. To perform a systematic comparison of FLT3 inhibitors, ten different FLT3 inhibitors with a wide range of selectivity towards FLT3 were included in the analysis (Supplemental Table 2). Cancer cell specific drug responses were quantified as selective drug sensitivity scores (sDSS) (DSS represents a modified area under the curve) for each inhibitor by comparing drug responses of the patients to 13 healthy controls using methods described in the Supplementary information. As expected, all FLT3 inhibitors had significantly higher sDSS showing greater efficacy in *FLT3*-ITD^+^ compared to *FLT3*-ITD^-^ samples. Interestingly, we also found that FLT3 inhibitor sensitivity was higher in the ITD-AR^high^ samples compared to the ITD-AR^low^ samples. Moreover, each FLT3 inhibitor had higher median sDSS in *FLT3*-ITD^+^ R/R samples compared to unmatched diagnostic *FLT3*-ITD^+^ samples (Fig. 1A, Supplemental Table 3, Fig. S4). The more prominent FLT3 inhibitor responses observed in the ITD-AR^high^ subgroup, consisting mostly of R/R samples, indicates clonal dominance of *FLT3*-ITD^+^ cells increase during disease progression.

To study whether the observed drug responses are associated with ITD-AR, ITD length, or blast count, we correlated the variables with each FLT3 inhibitor responses (Fig. 1B). As shown in Figs. S5–S7, we found strong positive correlation with ITD-AR and nine FLT3 inhibitors, moderate correlation with blast count and five FLT3 inhibitors, and no correlation between ITD length and FLT3 inhibitor response. The highest correlation with ITD-AR occurred with crenolanib and quizartinib. To examine whether *FLT3*-ITD allelic burden is directly associated with ex vivo FLT3 inhibitor responses, we performed orthogonal drug testing of *FLT3*-ITD^+^ (MOLM-13) and *FLT3*-ITD^-^ (Daudi) cell lines and their mixtures in varying proportions using a high-throughput flow cytometry-based DSRT-assay with five FLT3 inhibitors. By measuring relative viabilities of CD19 + (Daudi) and CD19- (MOLM-13) cells and their mixtures after 72 h treatment with five FLT3 inhibitors, we generated experimental evidence showing that increasing ITD-AR leads to stronger response to all five FLT3 inhibitors. Moreover, high sensitivity for specific FLT3 inhibitors guides the selection of highly specific or non-specific FLT3 inhibitors (e.g. gilteritinib and midostaurin) based on the *FLT3*-ITD-AR (Fig. 1C, Fig. S8). In the assay, dose-dependent viability reductions upon FLT3 inhibitor treatment were mainly observed in *FLT3*-ITD^+^ cells (CD19-), whereas the relative percentage of viable *FLT3*-ITD^-^ cells (CD19 +) remained unchanged or increased at higher drug concentrations. The strongest responses were observed for *FLT3*-ITD heterozygous MOLM-13 cells, whereas *FLT3*-ITD^-^ Daudi cells showed no response to FLT3 inhibitors other than non-selective multikinase inhibitor lestaurtinib, which showed off-target efficacy in *FLT3*-ITD^-^ cells (Fig. 1D, Supplemental Table 4). Taken together, our results from two complementary drug testing methods suggest that ITD-AR predicts ex vivo FLT3 inhibitor responses better than blast count or ITD length. The findings are consistent with the Cucchi et al. study reporting that gilteritinib significantly reduces colony-forming capacity of high compared to low ITD-AR or wild type *FLT3* AML samples^12^. Regardless of these findings, we acknowledge that ITD-AR alone cannot solely explain all variation in FLT3 inhibitor response. Few patients within the *FLT3*-ITD^+^ cohort lacked response to FLT3 inhibitors regardless of high ITD-AR (Fig. 1A). In our earlier work, we observed that in vitro drug responses of *FLT3*-ITD transduced BM cells from Balb/C mice are drastically different compared to the same cells co-transduced with *FLT3*-ITD and *NUP98-NSD1* gene fusion^9^, highlighting that co-occurring mutations also impact FLT3 inhibitor response.

**Figure 2:**
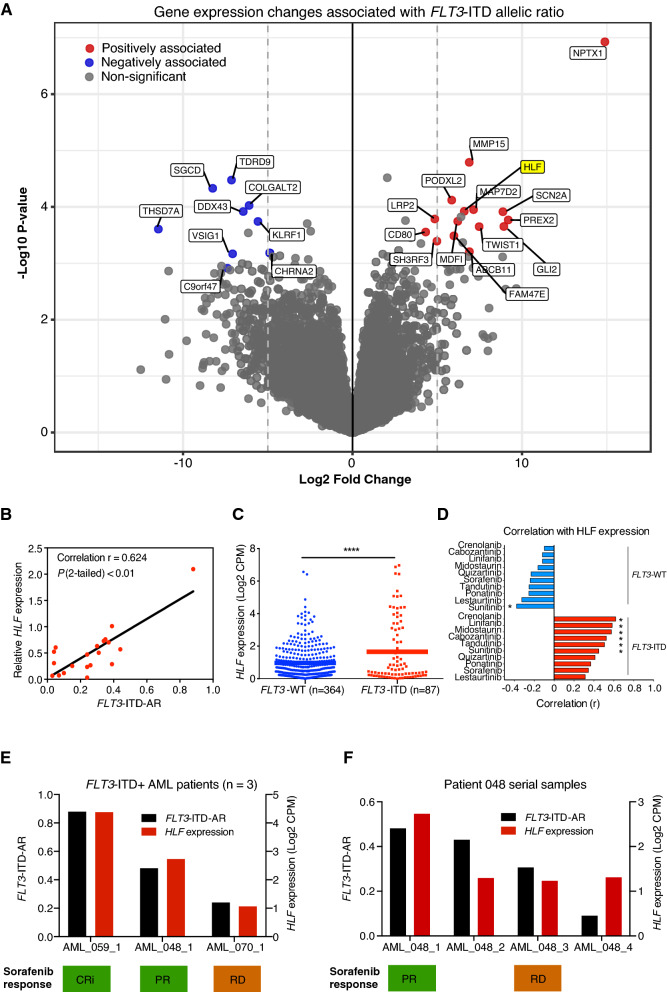
Gene expression profiles associated with *FLT3*-ITD-AR and FLT3 inhibitor response. (**A**) The volcano plot depicts protein coding genes (N = 14 141) with positive (red) or negative (blue) association with the ITD-AR identified by linear regression analysis of *FLT3*-ITD^+^ samples (N = 31). The genes with FDR < 0.1 and log2 fold change > 5 were considered significant. (**B**) qPCR validation experiment with 20 *FLT3*-ITD^+^ RNA samples confirmed association of *HLF* with ITD-AR. The figure shows correlation analysis of relative *HLF* quantity and ITD-AR levels. The *HLF* expression was normalized against four reference genes (*EIF4B*, *RPL19*, *SH3D19*, and *NACA*) with uniform expression across samples. (**C**) Expression of *HLF* was compared between *FLT3*-ITD^+^ and *FLT3*-WT patients using the BeatAML dataset. (**D**) The waterfall plot shows Pearson correlation coefficients between *HLF* gene expression (Log2 CPM) and selective FLT3 inhibitor responses in *FLT3-*WT and *FLT3*-ITD^+^ samples. Response to six inhibitors were associated (*P* < 0.05) with *HLF* expression. Significant associations are denoted with an asterisk (*, *P* < 0.05). (**E**, **F**) FLT3 inhibitor sorafenib was selected for the treatment of three chemorefractory AML patients based on ex vivo DSRT and molecular profiling. ITD-AR and *HLF* expression were retrospectively correlated with clinical response to sorafenib. The treatment outcomes were defined as complete remission with incomplete hematological recovery (CRi), partial remission (PR) and resistant disease (RD) evaluated based on ELN 2017 criteria.

To identify additional factors associated with ITD-AR and FLT3 inhibitor response, we next analyzed RNA-Seq gene expression data from 31 *FLT3*-ITD^+^ samples using methods described in the supplemental data and previously^13^. Linear regression analysis revealed 24 genes significantly associated with ITD-AR (FDR < 0.1 and log2 fold change >  ± 4). Positive association with ITD-AR was found for 15 genes and negative association for nine genes (Fig. 2A, Supplemental Table 5). To further support these findings, quantitative-PCR validation was performed on four of the associated genes, namely *HLF*, *KLRP1*, *MDFIC*, and *NPTX1,* using total RNA from 20 *FLT3*-ITD^+^ AML patients. The analysis confirmed significant association between Hepatic Leukemia Factor (*HLF*) and ITD-AR (r = 0.624, *P* < 0.01) (Fig. 2B), while no association with the other genes was observed (Supplemental Tables 6–7). As the validation cohort was unbalanced (14 diagnostic, 6 R/R), the same analysis was repeated for diagnostic samples only and showed comparable results. ITD-AR associated with *HLF* expression (r = 0.667, 95% CI 0.195–0.888, *P* = 0.011), but not with *KLRP1*, *MDFIC*, or *NPTX1* (Supplemental Table 7). We next compared *HLF* expression between *FLT3*-ITD^+^ (n = 87) and *FLT3* WT (n = 364) AML samples from the BeatAML dataset and found significantly (*P* < 0.0001) higher expression in the *FLT3*-ITD^+^ samples (Fig. 2C)^14^. *HLF* encodes a transcription factor that modulates fate of the hematopoietic lineage and was recently shown to regulate leukemic stem cells in triple-mutant AML with *FLT3*-ITD^+^, *NPM1,* and *DNMT3A*^15,16^. However, as our cohort included only one patient with the triple-mutant genotype, we could not study the exact mechanism of *HLF* mediated leukemic stem cell regulation in this subgroup of AML. The *FLT3*-ITD^+^ samples included in the gene expression analysis showed significant association between *HLF* expression and ex vivo response to crenolanib, midostaurin, sunitinib, tandutinib, cabozantinib, and linifanib, while *FLT3* WT samples showed no correlation (Fig. 2D, Supplemental Table 8). This finding suggests that ITD-AR together with associated *HLF* expression can predict ex vivo FLT3 inhibitor responses better compared to ITD-AR or *HLF* alone.

Our cohort included four chemorefractory AML patients treated with sorafenib based on DSRT results under off-label compassionate usage as part of a leukemia precision medicine program. Three patients had high ITD-AR and *HLF* expression whereas one had low ITD-AR (Supplemental Table 9). Clinical responses to sorafenib were observed in the patients with high ITD-AR and *HLF* expression, while the low ITD-AR and *HLF* expressing patient lacked response (Fig. 2E). Further analysis of ITD-AR and *HLF* expression in serial samples collected at different time points from patient 048 showed consistent results suggesting that ITD-AR associates with FLT3 inhibitor responses in vivo (Fig. 2F).

Taken together, the preliminary analyses of FLT3 inhibitor treated patients support that ITD-AR and associated *HLF* gene expression changes can improve the prediction FLT3 inhibitor efficacy in adult *FLT3*-ITD^+^ AML. The ex vivo results suggest that highly selective FLT3 inhibitors have more prominent responses in *FLT3*-ITD^+^ AML patients with high ITD-AR regardless of disease stage, while patients with low ITD-AR show stronger responses to non-selective kinase inhibitors targeting FLT3 (e.g. midostaurin). Based on the preclinical data, *FLT3*-ITD^+^ AML patients with high ITD-AR at diagnosis may respond well to gilteritinib, which currently lacks regulatory approval for newly diagnosed AML with FLT3 mutation. We suggest that *HLF* expression together with ITD-AR should be evaluated further as a potential dual-biomarker approach for treatment selection of *FLT3*-ITD^+^ AML.

### Ethics approval and consent to participate

The study was performed with the approval of Helsinki University Hospital Ethics Committee (permit numbers: 239/13/03/00/2010 and 303/13/03/01/2011) and in accordance with the Declaration of Helsinki. A written informed consent was acquired from all patients before sample collection.

## Supplementary Information

Supplementary Information.

## Abbreviations

AML: Acute myeloid leukemia
AR: Allelic ratio
BM: Bone marrow
CI: Confidence interval
DSRT: Drug sensitivity and resistance testing
DSS: Drug sensitivity score
FDR: False discovery rate
FLT3: FMS-like tyrosine kinase 3
HLF: Hepatic leukemia factor
ITD: Internal tandem duplication
qPCR: Quantitative polymerase chain reaction
R/R: Relapsed or refractory
sDSS: Selective drug sensitivity score
WT: Wild type
