# Supplementary material for: FLT3-ITD allelic ratio and HLF expression predict FLT3 inhibitor efficacy in adult AML
Source: Sci Rep. 2021 Dec 7;11:23565. doi: 10.1038/s41598-021-03010-7 (PMC8651734; doi:10.1038/s41598-021-03010-7)
Supplement: Supplementary file 1 — Supplementary Information. [file 41598_2021_3010_MOESM1_ESM.docx]

**Additional file 1**

***FLT3*-ITD allelic ratio and *HLF* expression predict FLT3 inhibitor efficacy in adult AML**

**Authors**: Jarno Kivioja,^1, *^ Disha Malani,^1, *^ Ashwini Kumar,^1^ Mika Kontro,^2,3^ Alun Parsons,^1^ Olli Kallioniemi,^1,4^ and Caroline A. Heckman^1^

**Affiliations:**

*^1^Institute for Molecular Medicine Finland – FIMM, HiLIFE - Helsinki Institute of Life Science, iCAN Digital Precision Cancer Medicine Flagship, University of Helsinki, Helsinki, Finland; ^2^Hematology Research Unit Helsinki, University of Helsinki; ^3^Department of Hematology, Helsinki University Hospital Comprehensive Cancer Center, Helsinki, Finland; ^4^Science for Life Laboratory, Department of Oncology and Pathology, Karolinska Institutet, Stockholm, Sweden.*

**Correspondence:**

Caroline A. Heckman, Ph.D.

Institute for Molecular Medicine Finland – FIMM, HiLIFE – Helsinki Institute of Life Science, University of Helsinki, P.O. Box 20 (Tukholmankatu 8), 00290 Helsinki, Finland;

Phone: +358 29 412 5769; e-mail: caroline.heckman@helsinki.fi

*: These authors contributed equally to this work.

**SUPPLEMENTARY METHODS**

**Patient samples**

The patient cohort consisted of 38 *FLT3*-ITD^+^ AML patients, 49 *FLT3*-ITD^-^ AML patients, and 13 healthy donors. Median age of the *FLT3*-ITD^+^ patients at the time of sampling was 58 years (range 29 – 78). Mononuclear cells (MNCs) were extracted from bone marrow (BM) aspirates or blood draws using Ficoll-Paque density gradient centrifugation (Ficoll-Paque Premium™, GE Healthcare, Little Chalfont, United Kingdom). After the extraction, MNCs were either viably frozen or suspended in freshly prepared Mononuclear Cell Medium (MCM, PromoCell, Heidelberg, Germany) supplemented with gentamicin (0.5 μg/ml) and amphotericin B (2.5 μg/ml). Demographic and clinical characteristics of the *FLT3*-ITD^+^ study patients are shown in Supplementary Table 1. The study was performed with the approval of Helsinki University Hospital Ethics Committee (permit numbers: 239/13/03/00/2010 and 303/13/03/01/2011) and in accordance with the Declaration of Helsinki. A written informed consent was acquired from all patients before sample collection.

**Fragment Analysis for *FLT3*-ITD**

To determine *FLT3*-ITD allelic ratio (mutant/total *FLT3*) and the length of ITD mutations, genomic DNA was extracted from MNCs of all study samples (N = 119) using DNeasy Blood and Tissue kit (Qiagen, Hilden, Germany; catalog number: 69506) or ALLPrep DNA/RNA Micro Kit (Qiagen; catalog number: 80284) following the manufacturer’s protocol. DNA concentrations were measured with the Qubit fluorometer (Thermo Fisher Scientific, Carlsbad, CA, USA). *FLT3*-ITD was PCR-amplified from 10 ng of genomic DNA with Platinum™ Taq DNA polymerase (2.5 Units per 50 μL reaction). 10X reaction buffer, MgCl_2_ (3 mM), dNTP mix (0.2 mM each), and primers 5′-6FAM GCAATTTAGGTATGAAAGCCAGC-3′ (forward) and 5′-CTTTCAGCATTTTGACGGCAACC-3′ (reverse). The PCR-conditions were as follows: initial denaturation at 94°C for 5 min, followed by 35 cycles at 94°C for 30 s, 66°C for 1 min, and 72°C for 2 min. The final annealing time at 72°C was set for 7 min. The amplified PCR products were run on a 2% agarose gel in the presence of SYBR® Safe DNA Gel Stain (Thermo Fisher Scientific) for 90 min at 90V. Subsequently, PCR products were diluted in sterile milli-Q water (1:100 and 1:200). Two microliters of each dilute was mixed with a reaction solution containing Hi-Di formamide and GeneScan™ 500 LIZ™ or 1200 LIZ™ (Thermo Fisher Scientific) dye size standard. The mix was added to barcoded MicroAmp ABI 96-well optical reaction plates (Thermo Fisher Scientific), centrifuged, and sealed prior to the analysis run on the ABI3730xl DNA analyzer (Applied Biosystems, Foster City, CA, USA). Each plate included several control wells with Hi-Di formamide alone, PCR-amplified FLT3 fraction from negative controls (*FLT3*-ITD^-^ patient sample), and milli-Q water in reaction mix. The mutant peaks (number, length, and area) were analyzed with GeneMapper software (Applied Biosystems). The *FLT3*-ITD allelic ratio (ITD-AR) was determined by comparing the area under the curve (AUC) of peaks corresponding to mutant and wild type alleles as well as the ratio of fluorescence peak heights (mutant/total *FLT3*) as described earlier (1). The median ITD-AR was calculated from all ITD^+^ samples, whereas the median ITD length was calculated from the ITD^+^ samples with one detectable ITD mutation (one sample/patient). The ITD length was determined by calculating the difference between the measured length of wild type *FLT3* fragment and the *FLT3*-ITD fragment.

**Drug sensitivity and resistance testing (DSRT)**

*Ex vivo* FLT3 inhibitor screening was performed using 78 samples from 54 adult AML patients and 13 healthy controls. The patient samples were collected at diagnosis (N = 29), relapse (N = 23), and refractory stage (N = 13). Briefly, ten FLT3 inhibitors with a wide range of target specificities were selected for the study including multi-kinase inhibitors (e.g. lestaurtinib) and agents with enhanced specificity to FLT3 (e.g. gilteritinib) (Supplementary Table 2). The compounds were plated onto tissue culture treated 384-well microplates (Corning, New York, NY, USA) in a series of five concentrations across a 10 000-fold concentration range using an Echo 550 acoustic dispenser (Labcyte Inc, Sunnyvale, CA, USA) as previously described (2). The prepared assay plates were stored in pressurized StoragePods® (Roylan Developments Ltd, Fetcham, United Kingdom) in the presence of inert nitrogen gas until use or a maximum of 5 weeks from the preparation. The compounds were first solubilized with 5 μl of MCM prior to adding 20 μl of cell suspension (10 000 cells/well) to each well with Multidrop™ Combi Reagent Dispenser (Thermo Fisher Scientific). Assay plates were agitated with Titramax 1000 platform shaker (Heidolph, Schwabach, Germany) for 5 min at 450 rpm between the liquid dispensing steps and briefly centrifuged to gather cells on the bottom of each well. Assay plates were incubated at +37°C for 72h. After the incubation, cell viabilities were measured by adding 25 μl of CellTiter-Glo® reagent (Promega, Madison, WI, USA) to each well and measuring luminescence with PHERAstar® FS microplate reader (BMG Labtech, Offenburg, Germany).

**Drug response data analysis**

The data quality and drug sensitivity score (DSS) of each FLT3 inhibitor was assessed from modified AUC as previously described (3). The selective DSS (sDSS) of each FLT3 inhibitor in the analyzed patient samples are shown in Supplementary Table 3. The sDSS values were calculated by subtracting the mean DSS of healthy BM control samples from patient samples (DSS^sample^ – DSS^control^). The samples and sDSS were clustered hierarchically using Euclidean distance matrix and complete clustering method. The drug-drug correlation map analysis was performed using the corrplot R package to display FLT3 inhibitors with similar efficacies.

**Flow cytometry-based DSRT**

Validation experiments were performed by high-throughput flow cytometric analysis using five FLT3 inhibitors with varying specificities towards FLT3 (gilteritinib, midostaurin, quizartinib, sorafenib, and lestaurtinib). FLT3 inhibitors were printed onto Nunc™ Microwell™ 96-Well V-bottom Microplates (Corning) in five concentrations (0.1, 1, 10, 100, and 1 000 nM) in duplicates using the Echo 550 acoustic dispenser (Labcyte). To confirm that *FLT3*-ITD-AR impacts FLT3 inhibitor responses, we treated MOLM-13 (*FLT3*-ITD^+^) and DAUDI (*FLT3*-ITD^-^) cell lines (100 000 cells/well) alone and in three co-culture mixtures (25/75, 50/50, and 75/25) with FLT3 inhibitors for 72h at +37°C. The cell lines were selected based on their *FLT3*-ITD mutational status, similar doubling time, and identical culturing conditions (RPMI-1640 with 10% FCS, 1% L-Glut, and 1% pencillin-streptomycin). The DAUDI cell line was acquired from the ATCC (ATCC® CCL-213™) and MOLM-13 from the DSMZ (catalog number: ACC 554). On day 0, both cell lines and their mixtures were single stained with CD19-APC (BD Pharmingen, Franklin Lakes, NJ, USA; catalog number: 555415) and Annexin V-PE (BD Pharmingen; catalog number: 556421) according to the manufacturer’s instructions and compared with the same cell lines or cell line mixtures stained with IgG1-APC (BD catalog number: 555751) and Annexin V-PE. Gates were set for viable CD19+ DAUDI cells and CD19- MOLM-13 cells and the percentage of CD19+ cells in each sample at the beginning of the experiment was analyzed. After 72h incubation at +37°C, 96-well plates were centrifuged at 600g for 5 min and flipped to remove culture medium. All staining reactions were done in a 100 µl reaction containing staining buffer (1xPBS + 0.5% BSA) and 8 ng of APC-conjugated antibody (CD19-APC or IgG1-APC) for 45 min on ice covered from light. After the staining, assay plates were centrifuged and flipped to remove staining solution. 100µl of Annexin V-PE staining mix (BD catalog number: 559763) was added to each well and incubated for 15 min prior to sample acquisition with the iQue PLUS screener (Sartorius, Göttingen, Germany). The viable cell counts in drug treated wells were normalized to responses from five positive (benzethonium chloride) and five negative (dimethyl sulfoxide) control wells to generate dose-response curves. The DSS of the five FLT3 inhibitors in each cell line and cell line mixture are shown in Supplementary Table 4.

**RNA sequencing and data analysis**

RNA was extracted from the MNC samples as previously described (2). The RNA integrity number (RIN) was determined with the 2100 Bioanalyzer using RNA 6000 Nano Kit (Agilent Technologies, Santa Clara, CA, USA). One to three micrograms high quality total RNA (RIN > 8) was ribo depleted and the RNA sequencing (RNA-Seq) library prepared using ScriptSeq v2 Complete kit (Illumina, San Diego, CA, USA). RNA-Seq libraries were purified with SPRI beads (Agencourt AMPure XP, Beckman Coulter, Brea, CA, USA). Subsequently, paired-end sequencing with 100 bp read length was performed with the HiSeq™2000 system (Illumina). The generated RNA-Seq data were preprocessed as previously described (4). Trimmomatic was used to correct read data for low quality, short read lengths, and Illumina adapters. Filtered reads were aligned to human genome (GRCh38) with STAR-aligner using EnsEMBL v82 gene models. The alignments were sorted and PCR duplicates marked using Picard. Feature counts were computed using SubRead and converted to expression estimates using Trimmed Mean of M-values normalization. Lowly expressed genomic features with counts per million values ≤ 1.00 were removed. Only protein-coding genes were considered for the analyses. A linear regression model was applied to identify genes associated with ITD-AR (Supplementary Table 5). In the linear regression model, RNA-Seq library preparation methods and RNA extraction kits were considered as major confounding factors.

**qPCR**

Expression of ITD-AR associated genes *HLF*, *NPTX1*, *MDFIC*, and *KLRF1* were validated for 20 patients by qPCR. The expression analysis was carried out on 10ng of cDNA prepared from total RNA using FIREScript RT cDNA Synthesis KIT following manufacturer’s instructions (Solis BioDyne, Tartu, Estonia). The qPCR reactions were performed using iQ SYBR Green Super Mix (Bio-Rad, Hercules, CA, USA) and a CFX384 Real-Time System (Bio-Rad). Three technical replicates were included for all samples and standards. Reference genes (*EIF4B*, *RPL19*, *SH3D19*, and *NACA*) were chosen based on their uniform expression across samples and used for normalizing the expression of target genes. The gene expression was quantified based on calculated primer efficiencies using the Pfaffl method (5). The oligonucleotide primers were acquired from Sigma-Aldrich (Saint Louis, MI, USA). The primer sequences and relative target gene expression values are listed in Supplementary Tables 6-7, respectively.

**Exome sequencing**

Exome sequencing was performed to 55 AML patients using genomic DNA extracted from BM MNCs. Genomic DNA (1-3 µg) was sequenced with the NimbleGen SeqCap EZ v2 capture kit (Roche NimbleGen, Madison, WI, USA) using HiSeq™2500 system (Illumina) and analyzed as previously described (6, 7).

**FLT3 inhibitor treatment of *FLT3*-ITD^+^ AML patients**

Three chemorefractory *FLT3*-ITD^+^ patients were treated with sorafenib as part of leukemia precision medicine program, which represents a collaborative effort between Institute for Molecular Medicine Finland and Helsinki University Hospital Comprehensive Cancer Center (Supplementary Table 8). The off-label treatment decisions for chemorefractory patients were made based on *ex vivo* drug testing and molecular profiling of each individual patient. Patient AML_048 was treated twice with sorafenib on different time points. The treatment response outcomes were defined as complete remission, partial remission, complete remission with incomplete hematological recovery, or resistant disease based on European Leukemia Net criteria (8). *FLT3*-ITD fragment analysis and gene expression analyses were done retrospectively. **Statistical Analyses**

Statistical analyses were done using R studio (RStudio Inc, Boston, MA, USA) and GraphPad Prism v8 (GraphPad Software, La Jolla, CA, USA). Group-wise comparisons of DSS, *FLT3*-ITD-AR, *FLT3*-ITD length, leukemic blasts, and leukocyte counts were analyzed using the Mann-Whitney U test. Correlation analyses were performed using the Pearson or the Spearman’s rank correlation coefficient test. All statistical analyses were two-tailed and P-values < 0.05 were considered significant.

**Supplementary References**

1. Zwaan CM, Meshinchi S, Radich JP, Veerman AJ, Huismans DR, Munske L, et al. FLT3 internal tandem duplication in 234 children with acute myeloid leukemia: prognostic significance and relation to cellular drug resistance. Blood. 2003;102(7):2387-94.

2. Kivioja JL, Thanasopoulou A, Kumar A, Kontro M, Yadav B, Majumder MM, et al. Dasatinib and navitoclax act synergistically to target NUP98-NSD1(+)/FLT3-ITD(+) acute myeloid leukemia. Leukemia. 2018.

3. Pemovska T, Kontro M, Yadav B, Edgren H, Eldfors S, Szwajda A, et al. Individualized systems medicine strategy to tailor treatments for patients with chemorefractory acute myeloid leukemia. Cancer Discov. 2013;3(12):1416-29.

4. Ritchie ME, Phipson B, Wu D, Hu Y, Law CW, Shi W, et al. limma powers differential expression analyses for RNA-sequencing and microarray studies. Nucleic Acids Res. 2015;43(7):e47.

5. Pfaffl MW. A new mathematical model for relative quantification in real-time RT-PCR. Nucleic Acids Res. 2001;29(9):e45.

6. Koskela HL, Eldfors S, Ellonen P, van Adrichem AJ, Kuusanmaki H, Andersson EI, et al. Somatic STAT3 mutations in large granular lymphocytic leukemia. N Engl J Med. 2012;366(20):1905-13.

7. Eldfors S, Kuusanmaki H, Kontro M, Majumder MM, Parsons A, Edgren H, et al. Idelalisib sensitivity and mechanisms of disease progression in relapsed TCF3-PBX1 acute lymphoblastic leukemia. Leukemia. 2017;31(1):51-7.

8. Dohner H, Estey E, Grimwade D, Amadori S, Appelbaum FR, Buchner T, et al. Diagnosis and management of AML in adults: 2017 ELN recommendations from an international expert panel. Blood. 2017;129(4):424-47.

**SUPPLEMENTARY FIGURE LEGENDS**

**Supplemental Fig. S1. *FLT3*-ITD and BM blast count distributions in the study cohort. (A-B)** The graphs show ITD-AR and ITD lengths in 51 samples from 38 *FLT3*-ITD^+^ AML patients. Gray color indicates patients with two distinct ITD mutations. (**C**) BM leukemic blast cell percentages in 116 AML patient samples. (**D-E**) The scatter plots show that there was no significant correlation with ITD-AR and *FLT3*-ITD length or BM blast percentage in the study cohort. (**F**) Samples collected during disease progression from three patients indicate consistency in ITD lengths.

**Supplemental Fig. S2. The impact of ITD length and ITD-AR on clinical and demographic variables. (A-F)** Comparison of ITD allelic ratio and ITD lengths between males and females, patients below and above 65 years of age, and between diagnostic and R/R samples. **(G-J)** The blood leukocyte counts and BM blast cell percentages were compared in sample groups divided based on median ITD-AR (0.338) and ITD length (45 bp), respectively. The horizontal bar shows mean and error bars the standard deviation. Samples with several different ITD mutations (n = 6) were excluded from the ITD length analysis, while PB MNC samples (n = 5) were excluded from all analyses.

**Supplemental Fig. S3. Kaplan-Meier curves for *de novo* AML patients according to *FLT3*-ITD-mutational status, *FLT3*-ITD mutation size, and *FLT3*-ITD-AR.** (**A**) Comparison of overall survival (OS) between AML patients with *FLT3*-ITD and wild type *FLT3*. There were no significant differences in OS between the groups. (B) Comparison of OS in patients with *FLT3*-ITD^+^ AML who had either short (< 45 bp) or long ITD mutation (≥ 45 bp). No significant differences in OS were found between the groups. (C) OS in patients who had either low (≤ 0.338) or high (> 0.338) ITD-AR at diagnosis. The curves show a significantly different OS between the two groups (*P* = 0.024, HR: 3.632, 95% CI 1.332 to 27.10). The 3-year OS rate in ITD-AR^high^ group was 17% compared to 67% in the ITD-AR^low^ group. Analyses were carried out by log-rank tests.

**Supplemental Fig. S4. *Ex vivo* FLT3 inhibitor responses in adult AML. (A)** The box and whiskers plot show selective FLT3 inhibitor responses (sDSS) in BM MNCs of adult AML patients with (n = 23) and without *FLT3*-ITD mutation (n = 38) in comparison to healthy BM MNCs (n = 13). (**B**) The sDSS values were further compared between *FLT3*-ITD^+^ patients with either low or high *FLT3*-ITD allelic ratio (mutant/total *FLT3*), (**C**) samples collected at diagnosis and at R/R stage, and (**D**) *FLT3*-ITD^+^ patients with either long (≥ 45 bp) or short (< 45 bp) ITD mutation. The graphs show all data points with median and interquartile range. The significance was analyzed using non-parametric Mann-Whitney U tests. Significant differences are indicated with an asterisk. * *P* < 0.05; ***P* < 0.01; ****P* < 0.001; **** *P* < 0.0001.

**Supplemental Fig. S5. Scatter plot showing correlation analysis between *FLT3*-ITD mutant allelic ratio and *ex vivo* response of *FLT3*-ITD^+^ AML samples to ten FLT3 inhibitors**. The correlation coefficient R-scores and P-values are shown in each graph. The yellow color indicates *FLT3*-ITD^+^ patient samples and orange color the ITD^+^ samples with *FLT3-*TKD (tyrosine kinase domain) mutation.

**Supplemental Fig. S6.** **Scatter plot showing correlation analysis between leukemic blast percentage and *ex vivo* response of *FLT3*-ITD^+^ AML samples to ten FLT3 inhibitors.** The correlation coefficient R-scores and *P*-values for each inhibitor are shown in each graph. The yellow color indicates *FLT3*-ITD^+^ AML patient samples and orange color the ITD^+^ AML samples with a *FLT3-*TKD point mutation.

**Supplemental Fig. S7.** **Scatter plot showing correlation analysis between ITD mutation length and *ex vivo* response of *FLT3*-ITD^+^ AML samples to ten FLT3 inhibitors.** The correlation coefficient R-scores and *P*-values for each inhibitor are shown inside the graphs. The yellow color indicates *FLT3*-ITD^+^ AML patient samples and orange color the ITD^+^ AML samples with a *FLT3-*TKD mutation.

**Supplemental Fig. S8. Flow cytometry-based FLT3 inhibitor validation experiment.** The FLT3 inhibitor responses were measured from *FLT3*-ITD^-^ DAUDI cell line (left), *FLT3*-ITD^+^ MOLM-13 cell line (right), and from three co-cultures (75:25. 50:50. and 25:75) of the two cell lines. The cell lines and co-cultures were incubated for 72 h in a humidified incubator at +37°C with the drugs and subsequently analyzed with a high-throughput flow cytometer (iQue PLUS screener, Sartorius) using CD19-APC antibody to distinguish the cell lines from each other. (**A-B)** The expression of CD19-APC in each cell line and cell line mixture was measured before and after the treatment. **(C)** Flow cytometry-based drug screening was performed to MOLM-13 (*FLT3*-ITD heterozygous) and DAUDI (*FLT3* WT) cell lines and their mixtures using five FLT3 inhibitors in five concentrations across 10 000-fold concentration range. The cell viabilities and CD19 expression were measured after 72 h incubation as described in the supplementary materials and methods. The dose-response curves illustrate that FLT3-specific inhibitors, such as gilteritinib, only show prominent *ex vivo* responses when FLT3-ITD-mutational burden is high. On the contrary, the multi-target kinase inhibitor lestaurtinib (orange) shows response even in the absence of *FLT3*-ITD^+^ cells due to off-target effects.

**SUPPLEMENTARY FIGURES**

**Fig. S1.**

**Fig. S2.**

**Fig. S3.**

**Fig. S4.**

**Fig. S5.**

**
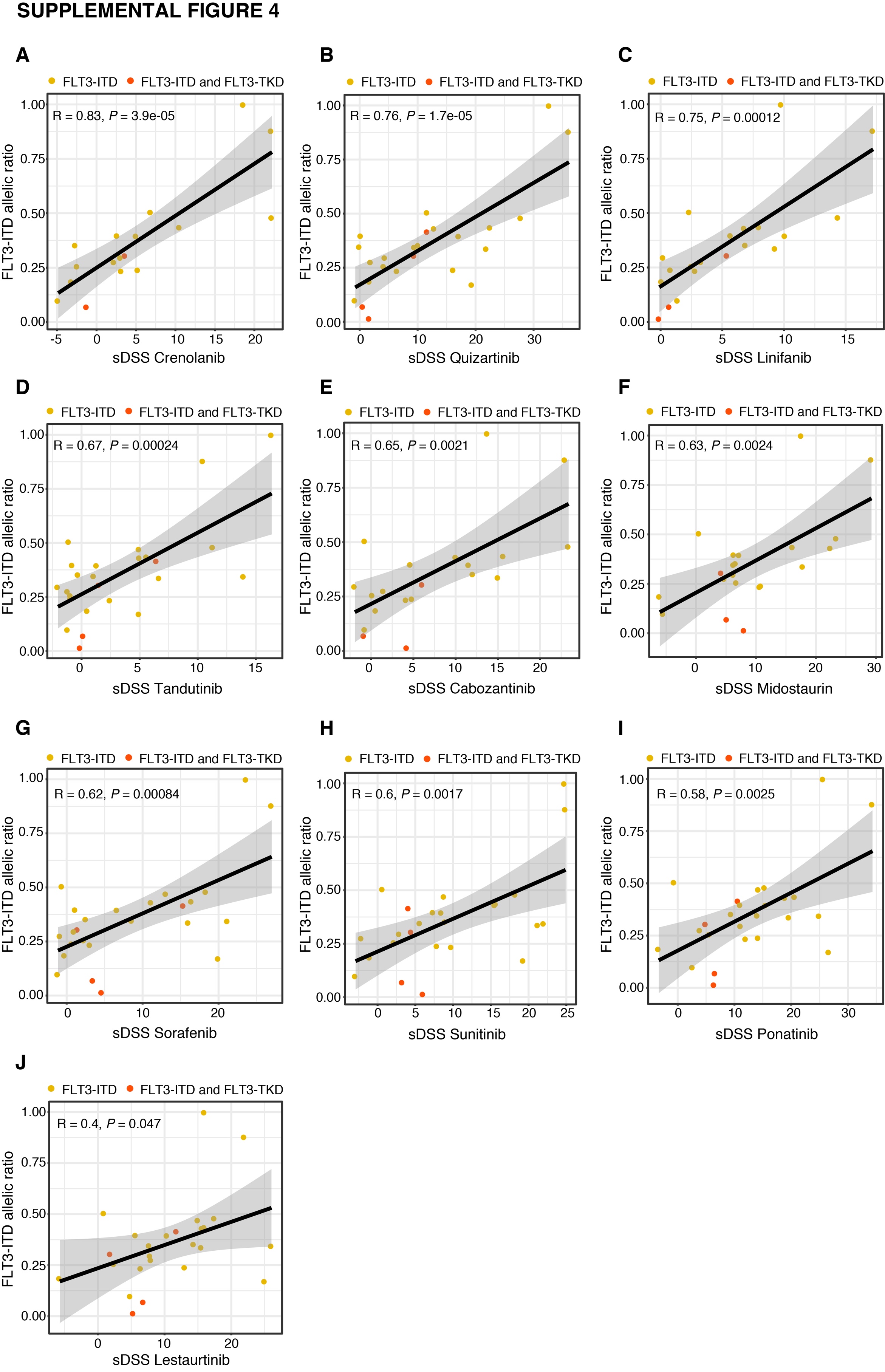
**

**Fig. S6.**


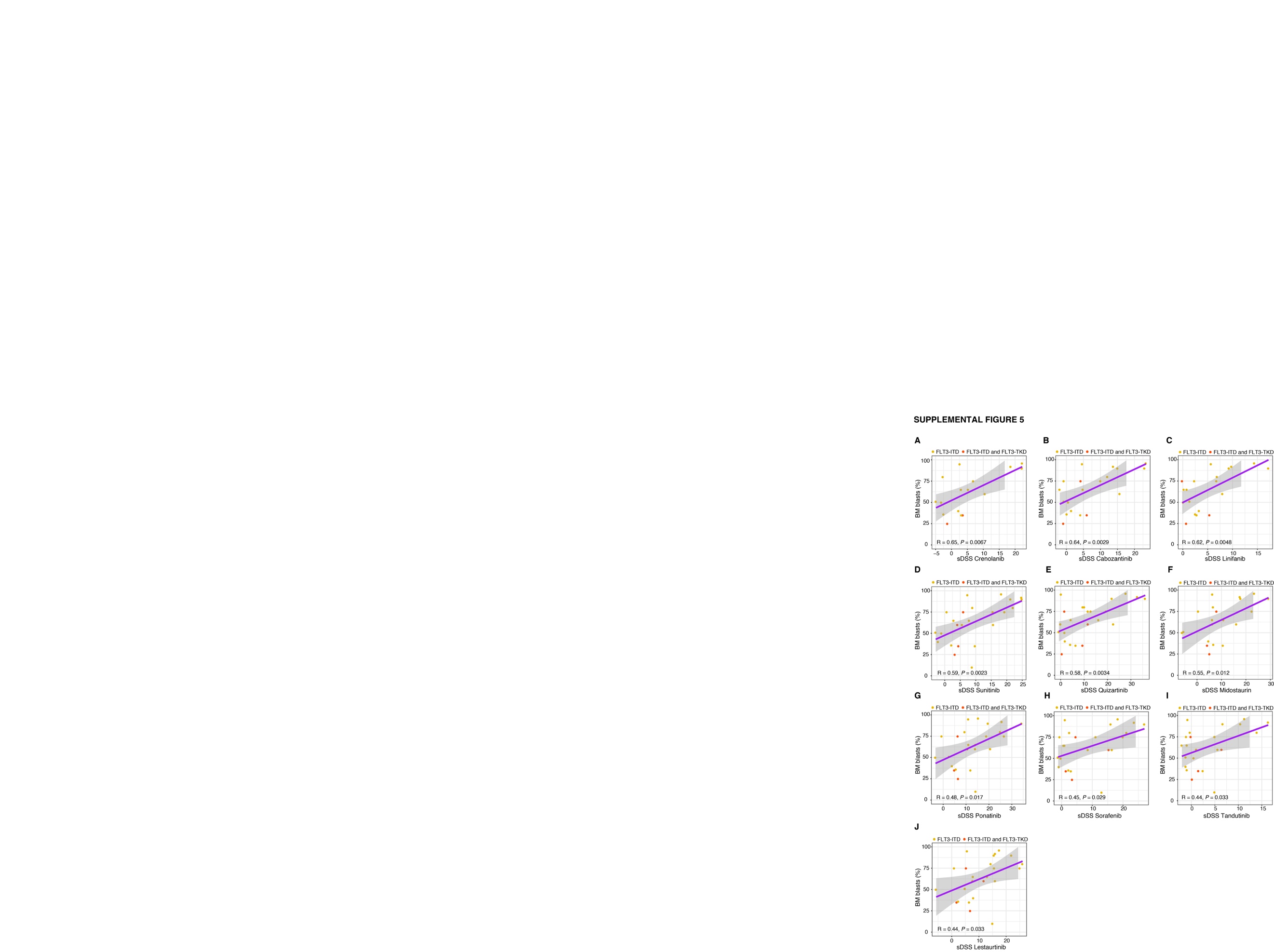


**Fig. S7.**


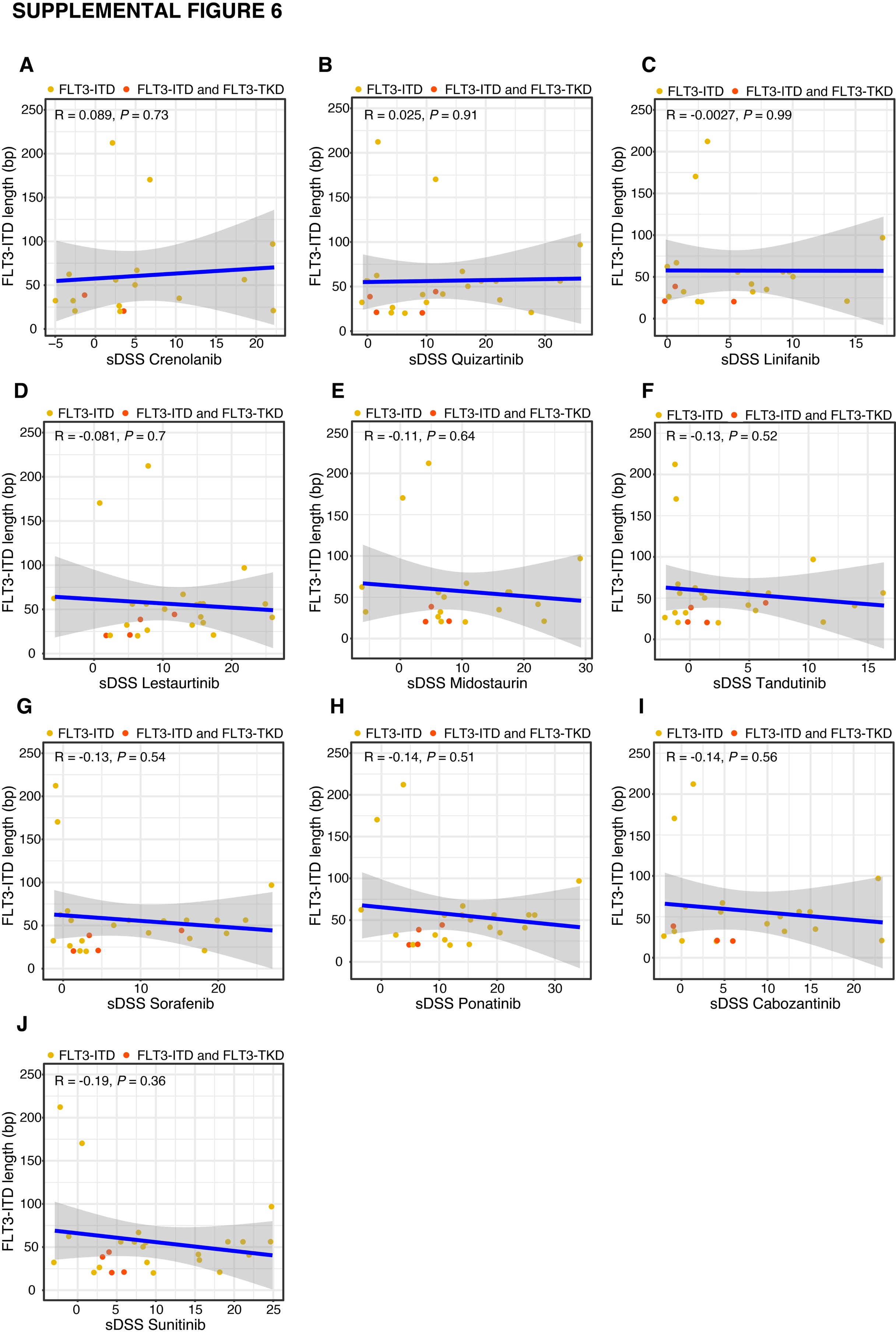


**Fig. S8.**

**
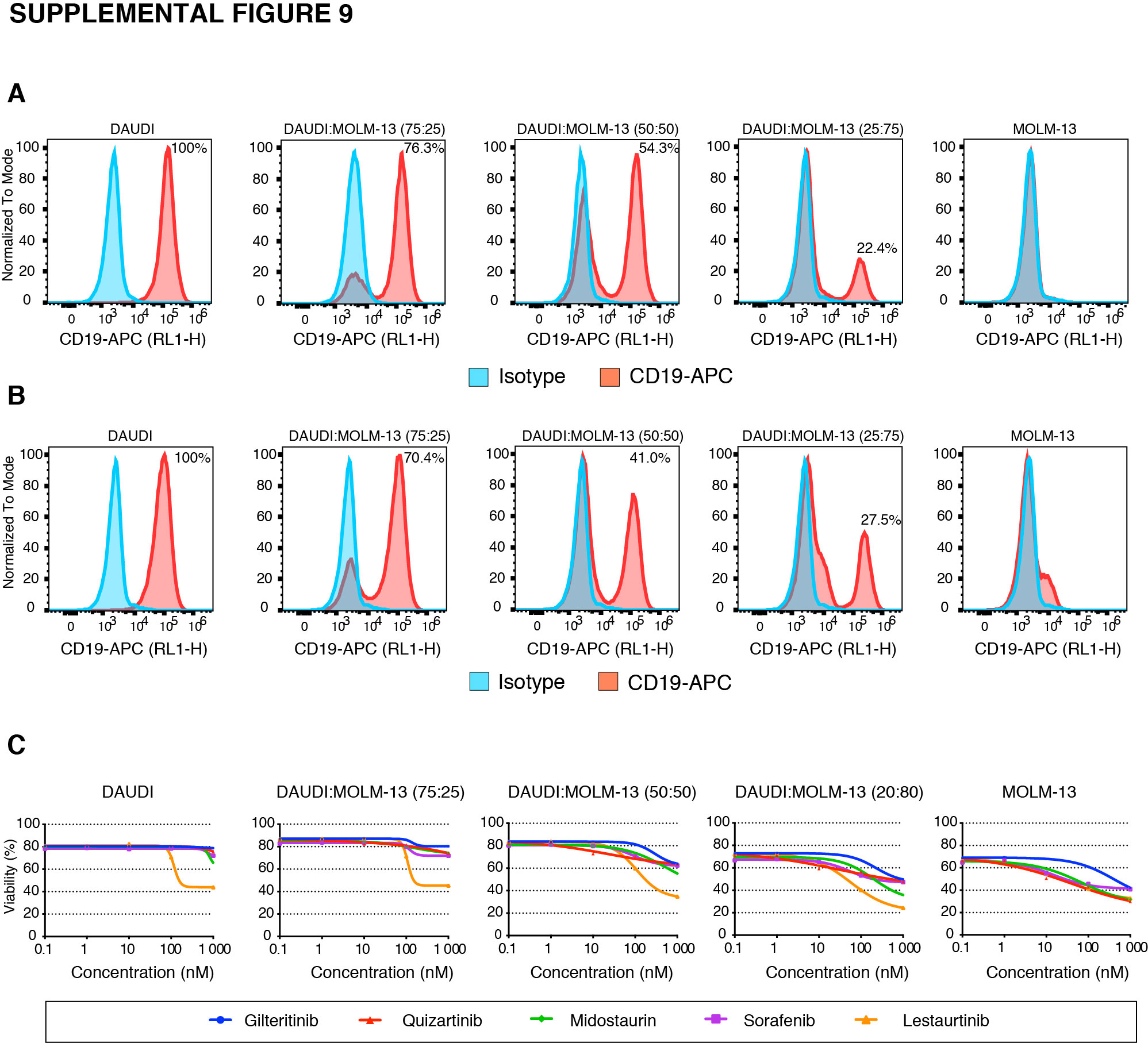
**

**SUPPLEMENTARY TABLES**

**Supplemental Table 1. Clinical and demographic characteristics of the patient cohort.**

|  | **Patient_ID** | **Sample_ID** | **Gender** | **Disease stage** | **Blast %** | **ITD-AR** | **ITD length (bp)** |
| --- | --- | --- | --- | --- | --- | --- | --- |
| ***FLT3*-ITD^+^ (N = 51)** | AML_002 | AML_002_1 | F | Relapse | 75 | 0.506 | 171 |
|  | AML_003 | AML_003_1 | F | Relapse | 92 | 1.000 | 57 |
|  | AML_005 | AML_005_3 | M | Relapse | 80 | 0.354 | 33 |
|  | AML_005 | AML_005_1 | M | Relapse | 51 | 0.099 | 33 |
|  | AML_007 | AML_007_1 | F | Relapse | 6 | 0.063 | 36 |
|  | AML_008 | AML_008_1 | F | Relapse | N/A | 0.828 | 17 |
|  | AML_009 | AML_009_1 | F | Refractory | 80 | 0.452 | 84 |
|  | AML_009 | AML_009_2 | F | Relapse | N/A | 0.309 | 84 |
|  | AML_011 | AML_011_2 | F | Refractory | 89 | 0.449 | 83 |
|  | AML_013 | AML_013_1 | M | Diagnosis | 82 | 0.370 | 33 |
|  | AML_014 | AML_014_1 | F | Diagnosis | 65 | 0.392 | 53 |
|  | AML_016 | AML_016_1 | M | Diagnosis | 74 | 0.031 | 67 |
|  | AML_018 | AML_018_1 | M | Diagnosis | 27 | 0.035 | 27 |
|  | AML_019 | AML_019_1 | M | Diagnosis | 55 | 0.043 | 74 |
|  | AML_024 | AML_024_1 | F | Diagnosis | 50 | 0.349 | 21 |
|  | AML_026 | AML_026_1 | M | Diagnosis | 65 | 0.297 | 27 and 39 |
|  | AML_027 | AML_027_1 | F | Diagnosis | 85 | 0.051 | 60 |
|  | AML_037 | AML_037_1 | F | Diagnosis | 90 | 0.338 | 57 |
|  | AML_038 | AML_038_1 | M | Relapse | 60 | 0.437 | 36 |
|  | AML_040 | AML_040_2 | M | Diagnosis | 23 | 0.073 | 45 |
|  | AML_045 | AML_045_4 | M | Refractory | 75 | 0.016 | 22 |
|  | AML_046 | AML_046_1 | F | Diagnosis | 55 | 0.452 | 48 |
|  | AML_048 | AML_048_1 | F | Refractory | 96 | 0.481 | 21 |
|  | AML_048 | AML_048_2 | F | Diagnosis | 65 | 0.430 | 21 |
|  | AML_048 | AML_048_3 | F | Relapse | 35 | 0.306 | 21 |
|  | AML_048 | AML_048_4 | F | Relapse | 35 | 0.090 | 21 |
|  | AML_053 | AML_053_1 | F | Diagnosis | 40 | 0.276 | 213 |
|  | AML_054 | AML_054_2 | M | Relapse | N/A | 0.451 | 55 |
|  | AML_054 | AML_054_1 | M | Relapse | 95 | 0.398 | 57 |
|  | AML_055 | AML_055_1 | M | Diagnosis | 36 | 0.257 | 21 |
|  | AML_059 | AML_059_1 | F | Relapse | 90 | 0.879 | 98 |
|  | AML_060 | AML_060_1 | M | Relapse | 82 | 0.390 | 42 |
|  | AML_062 | AML_062_1 | M | Relapse | 50 | 0.187 | 63 |
|  | AML_067 | AML_067_1 | M | Diagnosis | 25 | 0.071 | 39 |
|  | AML_070 | AML_070_2 | F | Refractory | 23 | 0.302 | 57 and 67 |
|  | AML_070 | AML_070_1 | F | Diagnosis | 65 | 0.240 | 68 |
|  | AML_073 | AML_073_1 | F | Diagnosis | 35 | 0.235 | 21 |
|  | AML_077 | AML_077_1 | M | Diagnosis | 90 | 0.451 | 27 |
|  | AML_078 | AML_078_1 | F | Relapse | 75 | 0.432 | 42 |
|  | AML_078 | AML_078_2 | F | Relapse | 80 | 0.346 | 42 |
|  | AML_078 | AML_078_3 | F | Relapse | 13 | 0.112 | 42 |
|  | AML_079 | AML_079_2 | M | Relapse | 10 | 0.472 | 57 and 62 |
|  | AML_079 | AML_079_3 | M | Relapse | 60 | 0.347 | 57 |
|  | AML_079 | AML_079_1 | M | Refractory | 75 | 0.172 | 57 |
|  | AML_080 | AML_080_1 | M | Diagnosis | 15 | 0.159 | 24 |
|  | AML_081 | AML_081_1 | F | Diagnosis | 60 | 0.189 | 99 |
|  | AML_082 | AML_082_2 | M | Relapse | 60 | 0.417 | 45 |
|  | AML_082 | AML_082_1 | M | Diagnosis | 35 | 0.335 | 45 |
|  | AML_083 | AML_083_1 | F | Refractory | 18 | 0.419 | 42 and 86 |
|  | AML_083 | AML_083_2 | F | Relapse | 14 | 0.237 | 42 and 87 |
|  | AML_084 | AML_084_1 | F | Diagnosis | 70 | 0.396 | 51 and 57 |
| ***FLT3*-ITD^-^ (N =68)** | AML_001 | AML_001_1 | M | Relapse | 30 | 0.000 |  |
|  | AML_004 | AML_004_1 | M | Relapse | 80 | 0.000 |  |
|  | AML_005 | AML_005_2 | M | Diagnosis | 72 | 0.000 |  |
|  | AML_006 | AML_006_1 | M | Relapse | 10 | 0.000 |  |
|  | AML_010 | AML_010_1 | F | Diagnosis | 64 | 0.000 |  |
|  | AML_011 | AML_011_1 | F | Diagnosis | 80 | 0.000 |  |
|  | AML_012 | AML_012_1 | F | Diagnosis | 60 | 0.000 |  |
|  | AML_015 | AML_015_1 | F | Diagnosis | 93 | 0.000 |  |
|  | AML_017 | AML_017_1 | M | Diagnosis | 60 | 0.000 |  |
|  | AML_020 | AML_020_1 | F | Diagnosis | 41 | 0.000 |  |
|  | AML_021 | AML_021_1 | F | Diagnosis | 75 | 0.000 |  |
|  | AML_022 | AML_022_1 | F | Diagnosis | 67 | 0.000 |  |
|  | AML_023 | AML_023_1 | F | Diagnosis | 92 | 0.000 |  |
|  | AML_025 | AML_025_1 | F | Diagnosis | 35 | 0.000 |  |
|  | AML_028 | AML_028_1 | F | Diagnosis | 70 | 0.000 |  |
|  | AML_029 | AML_029_1 | M | Diagnosis | 85 | 0.000 |  |
|  | AML_030 | AML_030_1 | F | Relapse | 22 | 0.000 |  |
|  | AML_030 | AML_030_2 | F | Relapse | 60 | 0.000 |  |
|  | AML_030 | AML_030_3 | F | Diagnosis | 50 | 0.000 |  |
|  | AML_031 | AML_031_1 | F | Diagnosis | 52 | 0.000 |  |
|  | AML_032 | AML_032_1 | M | Refractory | 9 | 0.000 |  |
|  | AML_032 | AML_032_2 | M | Refractory | 55 | 0.000 |  |
|  | AML_033 | AML_033_1 | F | Relapse | 45 | 0.000 |  |
|  | AML_034 | AML_034_1 | M | Relapse | 45 | 0.000 |  |
|  | AML_035 | AML_035_1 | M | Refractory | 40 | 0.000 |  |
|  | AML_035 | AML_035_2 | M | Diagnosis | 10 | 0.000 |  |
|  | AML_036 | AML_036_1 | F | Diagnosis | 60 | 0.000 |  |
|  | AML_039 | AML_039_1 | M | Diagnosis | 65 | 0.000 |  |
|  | AML_040 | AML_040_1 | M | Refractory | 75 | 0.000 |  |
|  | AML_040 | AML_040_3 | M | Refractory | 26 | 0.000 |  |
|  | AML_040 | AML_040_4 | M | Relapse | 46 | 0.000 |  |
|  | AML_040 | AML_040_5 | M | Refractory | 70 | 0.000 |  |
|  | AML_041 | AML_041_1 | M | Diagnosis | 39 | 0.000 |  |
|  | AML_042 | AML_042_1 | F | Diagnosis | 91 | 0.000 |  |
|  | AML_043 | AML_043_1 | M | Diagnosis | 85 | 0.000 |  |
|  | AML_044 | AML_044_1 | F | Relapse | 27 | 0.000 |  |
|  | AML_045 | AML_045_1 | M | Refractory | 65 | 0.000 |  |
|  | AML_045 | AML_045_2 | M | Refractory | 35 | 0.000 |  |
|  | AML_045 | AML_045_3 | M | Refractory | 40 | 0.000 |  |
|  | AML_047 | AML_047_1 | F | Diagnosis | 68 | 0.000 |  |
|  | AML_049 | AML_049_1 | F | Diagnosis | 30 | 0.000 |  |
|  | AML_050 | AML_050_1 | F | Refractory | 60 | 0.000 |  |
|  | AML_050 | AML_050_2 | F | Refractory | 95 | 0.000 |  |
|  | AML_051 | AML_051_1 | F | Diagnosis | 3 | 0.000 |  |
|  | AML_052 | AML_052_1 | M | Refractory | 25 | 0.000 |  |
|  | AML_056 | AML_056_1 | M | Refractory | 35 | 0.000 |  |
|  | AML_057 | AML_057_1 | F | Relapse | 55 | 0.000 |  |
|  | AML_057 | AML_057_2 | F | Refractory | 85 | 0.000 |  |
|  | AML_058 | AML_058_1 | F | Relapse | 25 | 0.000 |  |
|  | AML_061 | AML_061_1 | M | Relapse | 20 | 0.000 |  |
|  | AML_061 | AML_061_2 | M | Relapse | 20 | 0.000 |  |
|  | AML_063 | AML_063_1 | F | Diagnosis | 95 | 0.000 |  |
|  | AML_064 | AML_064_1 | M | Diagnosis | 85 | 0.000 |  |
|  | AML_065 | AML_065_1 | M | Diagnosis | 24 | 0.000 |  |
|  | AML_066 | AML_066_1 | F | Diagnosis | 60 | 0.000 |  |
|  | AML_068 | AML_068_1 | M | Diagnosis | 65 | 0.000 |  |
|  | AML_069 | AML_069_1 | M | Diagnosis | 50 | 0.000 |  |
|  | AML_071 | AML_071_1 | M | Diagnosis | 50 | 0.000 |  |
|  | AML_072 | AML_072_1 | M | Diagnosis | 66 | 0.000 |  |
|  | AML_074 | AML_074_1 | F | Diagnosis | 65 | 0.000 |  |
|  | AML_075 | AML_075_1 | M | Diagnosis | 25 | 0.000 |  |
|  | AML_076 | AML_076_1 | M | Relapse | 85 | 0.000 |  |
|  | AML_078 | AML_078_4 | F | Relapse | 5 | 0.000 |  |
|  | AML_081 | AML_081_2 | F | Relapse | 22 | 0.000 |  |
|  | AML_085 | AML_085_1 | F | Refractory | 65 | 0.000 |  |
|  | AML_085 | AML_085_2 | F | Refractory | 85 | 0.000 |  |
|  | AML_086 | AML_086_1 | F | Refractory | 55 | 0.000 |  |
|  | AML_087 | AML_087_1 | F | Diagnosis | 90 | 0.000 |  |

Footnotes: F, female; M, male; N/A, not available.

**Supplemental Table 2. FLT3 inhibitors included in the study.**

| **Drug name** | **Development phase for AML** | **Type** | **Additional targets** | **Supplier** | **Supplier Ref.** | **Solvent** | **Conc.**  **range (nM)** | **Drug testing method** |
| --- | --- | --- | --- | --- | --- | --- | --- | --- |
| Lestaurtinib | Discontinued | I | PKC, JAK2-3, TrkA, TrkB, TrkC, AurA-B | LC Laboratories | L-6307 | DMSO | 0.1-1 000 | CTG,  HT-FC |
| Sunitinib | Phase 1-2 | I | PDGFRB, KIT, RET, VEGFR2 | LC Laboratories | S-8803 | DMSO | 0.1-1 000 | CTG |
| Crenolanib | Phase 3 | I | PDGFRA/B, KIT | Selleck | S2730 | DMSO | 1-10 000 | CTG |
| Midostaurin | Approved | I | PDGFRB, KIT, PKC, Syk, Flk-1, Akt, PKA, FGR, SRC, VEGFR1/2 | LC Laboratories | P-7600 | DMSO | 1-10 000 | CTG,  HT-FC |
| Gilteritinib | Approved | I | LTK, AXL, ALK | ChemieTek | CT-GILT | DMSO | 0.1-1 000 | HT-FC |
| Tandutinib | Discontinued | II | PDGFRB, KIT | LC Laboratories | T-7802 | DMSO | 0.1-1 000 | CTG |
| Linifanib | Discontinued | II | VEGFR, PDGFRB, KDR, CSF-1R | Selleck | S1003 | DMSO | 0.1-1 000 | CTG |
| Cabozantinib | Phase 1 | II | VEGFR2, c-MET, TIE2, KIT, AXL, and RET | ChemieTek | CT-XL184 | DMSO | 0.1-1 000 | CTG |
| Ponatinib | Phase 2 | II | BCR-ABL1, KIT, LYN, PDGFRA, RET, SRC, VEGFR2, FGFR1-4 | Selleck | S1490 | DMSO | 0.1-1 000 | CTG |
| Sorafenib | Phase 1-3 | II | FGFR-1, VEGFR1-3, RET PDGFRB, KIT, RAF | LC Laboratories | S-8502 | DMSO | 0.1-1 000 | CTG,  HT-FC |
| Quizartinib | Phase 3 | II | PDGFRB, KIT, RET | ChemieTek | CT-AC220 | DMSO | 0.1-1 000 | CTG,  HT-FC |

Footnotes: CTG, Cell-Titer Glo; HT-FC, High-throughput flow cytometry.

**Supplemental Table 3. Selective FLT3 inhibitor responses (sDSS) in 65 AML samples compared to 13 healthy controls.**

| **Sample_ID** | **Tandutinib** | **Lestaurtinib** | **Quizartinib** | **Midostaurin** | **Sorafenib** | **Ponatinib** | **Crenolanib** | **Linifanib** | **Cabozantinib** | **Sunitinib** |
| --- | --- | --- | --- | --- | --- | --- | --- | --- | --- | --- |
| AML_002_1 | -1.1 | 1.0 | 11.7 | 0.5 | -0.6 | -0.6 | 6.9 | 2.4 | -0.7 | 0.7 |
| AML_003_1 | 16.4 | 16.0 | 32.7 | 17.6 | 23.7 | 25.7 | 18.6 | 9.8 | 13.8 | 24.8 |
| AML_004_1 | 1.7 | 9.3 | 5.3 | -2.2 | 2.7 | 11.2 | 5.5 | -0.3 | -0.8 | 3.0 |
| AML_005_1 | -1.2 | 4.9 | -0.8 | -5.5 | -1.1 | 2.7 | -4.9 | 1.4 | -0.7 | -2.9 |
| AML_005_2 | -1.2 | 4.8 | -0.8 | -1.0 | -1.1 | 6.3 | -3.0 | 0.4 | 0.6 | -1.5 |
| AML_005_3 | -0.3 | 14.4 | 10.1 | 6.6 | 2.6 | 9.5 | -2.7 | 6.9 | 12.1 | 9.0 |
| AML_006_1 | -1.2 | 2.2 | -0.3 | -0.9 | -1.1 | 2.5 | 0.2 | 1.6 | 2.3 | 1.0 |
| AML_026_1 | -2.0 | 7.9 | 4.4 | 6.3 | 1.0 | 11.1 | 3.1 | 0.2 | -1.9 | 2.9 |
| AML_030_1 | -1.2 | 8.0 | 2.0 | 5.4 | -1.1 | 4.6 | 1.7 | 0.0 | -0.7 | -2.2 |
| AML_030_2 | -1.2 | 8.3 | -0.7 | 2.7 | -1.1 | 6.0 | 1.3 | 0.1 | -0.7 | -1.1 |
| AML_030_3 | -0.4 | -0.4 | 0.0 | -2.2 | 0.9 | 1.9 |  |  |  | -3.0 |
| AML_031_1 | 2.4 | 9.2 | 2.0 | 0.3 | 6.1 | 8.7 |  |  |  | 1.7 |
| AML_032_2 | -1.2 | 7.6 | -0.8 | 0.5 | -1.1 | 0.9 | -3.2 | 1.3 | -0.7 | 1.4 |
| AML_033_1 | -1.9 | 8.7 | 2.0 | 4.0 | 0.3 | 3.8 | 0.8 | 1.8 | 2.4 | 1.9 |
| AML_034_1 | -0.3 | 3.8 | 0.0 | 0.1 | -0.1 | 2.9 |  | 0.7 | -0.2 | 0.5 |
| AML_035_1 | -0.3 | 6.4 | 0.0 | 2.1 | -0.1 | 1.6 |  | -0.1 | -0.2 | -1.7 |
| AML_035_2 | -0.3 | 7.6 | 0.0 | 5.8 | -0.1 | 2.0 |  | -0.1 | -0.2 | -0.3 |
| AML_036_1 | 1.5 | 3.8 | 0.0 | 4.1 | 2.3 | 3.2 |  | 0.6 | -0.2 | 1.8 |
| AML_037_1 | 6.7 | 15.6 | 21.9 | 17.9 | 16.1 | 19.7 |  | 9.3 | 15.1 | 21.3 |
| AML_038_1 | 5.6 | 16.0 | 22.5 | 16.1 | 16.5 | 20.7 | 10.5 | 8.0 | 15.7 | 15.7 |
| AML_039_1 | 0.2 | 9.0 | 0.0 | 11.2 | -0.1 | 6.7 |  | -0.1 | 0.1 | 5.5 |
| AML_043_1 | -1.0 | -5.7 | 0.6 | -6.1 | -1.1 | 1.0 | -3.2 | 0.0 | -0.7 | 2.9 |
| AML_044_1 | 15.4 | 27.3 | 6.5 |  | 4.5 | 10.0 |  |  |  | 9.9 |
| AML_045_4 | -0.1 | 5.4 | 1.7 | 8.1 | 4.7 | 6.5 |  | -0.1 | 4.3 | 6.1 |
| AML_047_1 | 3.5 | 9.7 | 4.7 | 9.4 | 0.9 | 10.9 | -4.5 | 0.0 | 10.2 | 9.9 |
| AML_048_1 | 11.3 | 17.5 | 27.8 | 23.4 | 18.4 | 15.4 | 22.2 | 14.4 | 23.4 | 18.3 |
| AML_048_3 | 1.5 | 1.9 | 9.4 | 4.2 | 1.5 | 5.0 | 3.6 | 5.4 | 6.1 | 4.5 |
| AML_049_1 | -1.2 | 2.9 | 2.3 | 0.3 | -1.1 | -1.6 | -0.5 | 0.0 | -0.7 | -3.7 |
| AML_050_1 | -1.2 | 2.9 | -0.8 | 5.6 | -1.1 | -2.8 | 1.8 | 0.0 | -0.7 | -3.9 |
| AML_050_2 | -1.2 | 3.5 | -0.8 | 4.7 | -1.1 | -2.8 | 2.5 | 0.0 | -0.7 | -3.9 |
| AML_051_1 | -1.2 | 4.8 | -0.8 | 0.5 | -1.1 | -0.1 | 0.2 | 0.0 | -0.7 | -3.9 |
| AML_052_1 | -1.2 | -3.2 | -0.8 | -6.1 | -1.1 | -3.5 | -3.7 | 0.0 | -0.7 | -3.9 |
| AML_053_1 | -1.2 | 8.0 | 1.9 | 4.7 | -0.8 | 4.0 | 2.2 | 3.3 | 1.5 | -2.1 |
| AML_054_1 | -0.8 | 5.7 | 0.2 | 6.3 | 1.2 | 11.1 | 2.6 | 5.7 | 4.7 | 7.4 |
| AML_055_1 | -0.9 | 2.5 | 4.2 | 6.8 | 2.3 | 5.6 | -2.4 | 2.5 | 0.2 | 2.2 |
| AML_056_1 | -2.0 | 5.1 | -0.2 | -2.1 | 0.0 | 4.4 | -4.9 | 1.5 | 1.3 | 5.1 |
| AML_057_1 | -0.7 | -1.4 | 2.8 | -6.1 | -0.4 | -0.7 | -4.9 | 0.2 | 1.6 | -1.8 |
| AML_058_1 | -1.2 | -3.5 | -0.8 | -3.0 | -1.1 | -2.5 | -4.1 | 0.0 | -0.7 | -3.3 |
| AML_059_1 | 10.5 | 22.0 | 36.1 | 29.3 | 27.0 | 34.3 | 22.2 | 17.2 | 23.0 | 24.9 |
| AML_061_1 | -0.4 | 16.9 | 0.0 | -2.1 | 1.5 | 17.3 |  |  |  | 0.8 |
| AML_061_2 | -0.4 | 11.4 | 0.0 | 1.1 | -0.5 | 8.5 |  |  |  | -1.9 |
| AML_062_1 | 0.5 | -5.7 | 1.7 | -6.1 | -0.2 | -3.3 | -3.2 | 0.1 | 0.6 | -1.0 |
| AML_063_1 | -1.2 | 4.5 | -0.8 | 0.3 | -1.1 | 0.7 | 0.7 | 2.0 | 4.1 | 1.0 |
| AML_064_1 | 1.6 | 9.1 | 11.8 | -6.1 | 4.9 | 8.9 | -4.6 | 3.6 | 11.2 | 8.5 |
| AML_065_1 | 0.7 | 8.7 | 0.3 | 4.7 | 0.0 | 6.9 | 1.4 | -0.5 | 1.4 | 3.4 |
| AML_066_1 | -1.8 | 3.5 | 2.1 | 4.2 | 1.3 | 2.3 | -2.6 | 4.0 | -1.1 | 4.3 |
| AML_067_1 | 0.2 | 6.9 | 0.6 | 5.2 | 3.5 | 6.6 | -1.2 | 0.7 | -0.8 | 3.3 |
| AML_068_1 | -1.8 | 2.1 | 2.8 | -2.2 | 0.8 | 4.1 | -4.9 | 1.1 | -2.0 | -0.4 |
| AML_069_1 | -2.0 | 4.6 | 5.1 | 0.4 | 0.0 | 3.0 | -3.1 | -0.5 | -2.2 | 2.0 |
| AML_070_1 | -0.9 | 13.1 | 16.2 | 10.9 | 0.7 | 14.2 | 5.3 | 0.8 | 4.9 | 7.9 |
| AML_071_1 | -2.0 | 5.5 | -1.0 | -1.2 | 0.0 | 5.2 | -0.8 | -0.4 | -1.4 | 0.6 |
| AML_072_1 | 1.2 | 11.7 | 6.6 | 9.7 | 1.2 | 12.8 | -0.4 | 2.2 | 2.3 | 5.0 |
| AML_073_1 | 2.5 | 6.5 | 6.5 | 10.7 | 3.1 | 12.0 | 3.2 | 2.8 | 4.2 | 9.8 |
| AML_074_1 | -2.0 | -3.9 | 2.2 | -2.2 | 0.0 | 0.0 | -4.9 | -0.5 | -2.9 | -1.5 |
| AML_075_1 | -1.5 | 3.8 | 1.8 | 3.6 | 0.5 | 4.0 | -5.2 | 0.0 | -2.3 | 1.8 |
| AML_078_1 | 5.0 | 15.7 | 12.9 | 22.5 | 11.2 | 19.0 |  | 6.8 | 10.1 | 15.6 |
| AML_078_2 | 14.0 | 26.0 | 9.5 |  | 21.2 | 25.0 |  |  |  | 22.0 |
| AML_079_1 | 5.0 | 25.0 | 19.4 |  | 20.0 | 26.7 |  |  |  | 19.3 |
| AML_079_2 | 5.0 | 15.0 |  |  | 13.1 | 14.3 |  |  |  | 8.8 |
| AML_079_3 | 1.1 | 7.7 | 0.0 | 6.3 | 8.6 | 14.1 |  |  |  | 5.6 |
| AML_081_2 | -1.2 | -5.7 | -0.8 | -3.2 | -1.1 | -2.0 | -0.6 | 0.0 | -0.7 | -2.9 |
| AML_082_2 | 6.5 | 11.8 | 11.7 |  | 15.4 | 10.7 |  |  |  | 4.1 |
| AML_084_1 | 1.3 | 10.4 | 17.1 | 7.2 | 6.7 | 15.6 | 5.0 | 10.1 | 11.6 | 8.5 |
| AML_085_1 | 2.0 | 13.1 | 0.0 |  | -0.8 | 4.9 |  |  |  | 7.7 |
| AML_087_1 | -0.4 | 19.6 | 6.8 | 9.2 | 5.9 | 13.7 |  |  |  | 5.6 |
| Control_01 | 0.6 | 2.1 | 0.0 | 0.0 | 1.3 | 4.9 |  |  |  |  |
| Control_02 | -0.1 | -1.3 | 0.0 |  | -2.7 | -0.3 |  |  |  |  |
| Control_03 | -0.4 | -0.9 |  |  | 1.4 | -4.5 |  |  |  |  |
| Control_04 | 0.3 | 0.0 | 0.0 | 1.2 | 0.1 | 0.3 |  | 0.1 | 0.2 | 0.3 |
| Control_05 | -0.3 | 0.1 | 0.0 | -1.2 | -0.1 | -0.3 |  | -0.1 | -0.2 | -0.3 |
| Control_06 | -1.2 | -1.4 | -0.3 | 3.3 | -0.9 | 1.4 | -0.9 | 0.0 | 2.9 | -0.3 |
| Control_07 | -0.7 | -0.3 | -0.8 | -2.0 | -0.5 | -0.9 | 1.2 | 0.0 | -0.7 | -1.8 |
| Control_08 | 4.2 | 2.9 | 2.5 | 1.7 | 3.8 | 2.3 | 0.1 | 0.0 | -0.7 | 3.1 |
| Control_09 | -1.2 | 3.8 | -0.8 | 2.9 | -1.1 | 1.5 | 1.6 | 0.0 | -0.7 | 1.5 |
| Control_10 | -1.2 | -5.0 | -0.8 | -6.1 | -1.1 | -4.1 | -2.2 | 0.0 | -0.7 | -2.4 |
| Control_11 | -0.1 | 1.6 | -0.2 | 2.6 | 0.0 | 0.1 | -0.3 | -0.5 | 0.7 | -0.9 |
| Control_12 | 0.2 | -0.8 | 0.5 | -2.2 | 0.0 | -0.2 | -0.1 | -0.3 | -0.9 | 0.3 |
| Control_13 | 0.0 | -0.7 | -0.3 | -0.3 | 0.0 | 0.0 | 0.5 | 0.7 | 0.3 | 0.6 |

**Supplemental Table 4. Drug sensitivity scores (DSS) of MOLM-13 (CD19-) and DAUDI (CD19+) cell lines and their co-cultures.** The validation experiment was done using high-throughput flow cytometry-based DSRT-assay to evaluate the utility of non-selective and selective FLT3 inhibitors in samples with variable FLT3-ITD mutational burden.

|  | **Lestaurtinib** | **Midostaurin** | **Sorafenib** | **Quizartinib** | **Gilteritinib** |
| --- | --- | --- | --- | --- | --- |
| DAUDI | 9.1 | 2.0 | 0.7 | 0 | 0.1 |
| DAUDI-MOLM-13 (75:25) | 8.5 | 1.1 | 0.6 | 0.5 | 0 |
| DAUDI-MOLM-13 (50:50) | 11.2 | 5.5 | 4.3 | 5.1 | 3.9 |
| DAUDI-MOLM-13 (25:75) | 14.0 | 6.7 | 5.8 | 6.0 | 4.8 |
| MOLM-13 | 17.3 | 14.6 | 17.5 | 17.0 | 6.2 |

**Supplemental Table 5. Gene expression profiles associated with *FLT3*-ITD allelic ratio.**

| **ENSEMBL ID** | **Gene** | **Gene description** | **Type** | **Log FC** | **FDR** | **Association with ITD-AR** |
| --- | --- | --- | --- | --- | --- | --- |
| ENSG00000171246 | NPTX1 | neuronal pentraxin 1 | Other | 14.96267 | 0.00000 | Positive |
| ENSG00000046889 | PREX2 | phosphatidylinositol-3.4.5-trisphosphate dependent Rac exchange factor 2 | Other | 9.18295 | 0.00017 | Positive |
| ENSG00000074047 | GLI2 | GLI family zinc finger 2 | Transcription regulator | 9.01468 | 0.00022 | Positive |
| ENSG00000136531 | SCN2A | sodium voltage-gated channel alpha subunit 2 | Ion channel | 8.94312 | 0.00012 | Positive |
| ENSG00000122691 | TWIST1 | twist family bHLH transcription factor 1 | Transcription regulator | 7.40666 | 0.00022 | Positive |
| ENSG00000184368 | MAP7D2 | MAP7 domain containing 2 | Other | 7.12240 | 0.00011 | Positive |
| ENSG00000102996 | MMP15 | matrix metallopeptidase 15 | Peptidase | 6.90592 | 0.00002 | Positive |
| ENSG00000073734 | ABCB11 | ATP binding cassette subfamily B member 11 | Transporter | 6.84692 | 0.00062 | Positive |
| ENSG00000108924 | HLF | HLF. PAR bZIP transcription factor | Transcription regulator | 6.50573 | 0.00012 | Positive |
| ENSG00000112559 | MDFI | MyoD family inhibitor | Other | 6.29848 | 0.00018 | Positive |
| ENSG00000189157 | FAM47E | family with sequence similarity 47 member E | Other | 5.97017 | 0.00033 | Positive |
| ENSG00000114631 | PODXL2 | podocalyxin like 2 | Other | 5.81074 | 0.00008 | Positive |
| ENSG00000172985 | SH3RF3 | SH3 domain containing ring finger 3 | Enzyme | 4.93943 | 0.00041 | Positive |
| ENSG00000081479 | LRP2 | LDL receptor related protein 2 | Transporter | 4.93740 | 0.00016 | Positive |
| ENSG00000121594 | CD80 | CD80 molecule | Transmembrane receptor | 4.38828 | 0.00028 | Positive |
| ENSG00000120903 | CHRNA2 | cholinergic receptor nicotinic alpha 2 subunit | Transmembrane receptor | -4.94025 | 0.00066 | Negative |
| ENSG00000150045 | KLRF1 | killer cell lectin like receptor F1 | Transmembrane receptor | -5.60408 | 0.00018 | Negative |
| ENSG00000198756 | COLGALT2 | collagen beta(1-O)galactosyltransferase 2 | Enzyme | -6.17592 | 0.00009 | Negative |
| ENSG00000080007 | DDX43 | DEAD-box helicase 43 | Enzyme | -6.48613 | 0.00012 | Negative |
| ENSG00000101842 | VSIG1 | V-set and immunoglobulin domain containing 1 | Other | -7.16497 | 0.00068 | Negative |
| ENSG00000156414 | TDRD9 | tudor domain containing 9 | Enzyme | -7.23335 | 0.00003 | Negative |
| ENSG00000186354 | C9orf47 | chromosome 9 open reading frame 47 | Other | -7.47451 | 0.00122 | Negative |
| ENSG00000170624 | SGCD | sarcoglycan delta | Other | -8.25039 | 0.00005 | Negative |
| ENSG00000005108 | THSD7A | thrombospondin type 1 domain containing 7A | Other | -11.54503 | 0.00025 | Negative |

Footnotes: FC, fold change; FDR, false discovery rate.

**Supplemental Table 6. Primer sequences used for qPCR.**

| Primer ID | Sequence (5'-> 3') | NCBI Accession number | Product size (bp) |
| --- | --- | --- | --- |
| 1566-KLRF1-F | GAAAAGGAGTTCTGCCCAAA | NM_016523.3 | 140 |
| 1567-KLRF1-R | CCAACAGGATCAAGGAGATCA |  |  |
| 1564-MDFIC-F | GGAGAGATGCAAGACCAGTCC | NM_199072.4 | 118 |
| 1565-MDFIC-R | TTTCCTCACCACTTGGCACC |  |  |
| 1559-NPTX1-F | CGAGCTCGAGAAAGGTCAGA | NM_002522.4 | 138 |
| 1561-NPTX1-R | AGCCACATGCAGACAGTGAA |  |  |
| 1561-HLF-F | CTTCACCCTGGCATCCCATC | NM_002126.5 | 125 |
| 1563-HLF-R | TAACCCACTGGGACCTGGAT |  |  |
| EIF4B-qPCR-F | GACGGGATGATCGGTCGTG | NM_001417.7 | 150 |
| EIF4B-qPCR-R | ACTGGGAGGTACTAGCAGAGG |  |  |
| RPL19-qPCR-F | ACATGGGCATAGGTAAGCGG | NM_000981.4 | 89 |
| RPL19-qPCR-R | CCGGCGCAAAATCCTCATTC |  |  |
| SH3D19-qPCR-F | AACCACCCAGGTCAAACAGG | NM_001009555.4 | 143 |
| SH3D19-qPCR-R | GTTGCAGAAAGCTTTGGGGG |  |  |
| NACA-qPCR-F | TCCCTTCCTTCAGAAATGCCCG | NM_001113201.3 | 150 |
| NACA-qPCR-R | CTGGGTGGAATCCTGTTCTTCA |  |  |

**Supplemental Table 7. Relative expression of *HLF*, *KLRF1*, *MDFIC*, and *NPTX1* in *FLT3*-ITD^+^ AML samples.**

|  |  | ***HLF*** | | | ***KLRF1*** | | | ***MDFIC*** | | | ***NPTX1*** | | |
| --- | --- | --- | --- | --- | --- | --- | --- | --- | --- | --- | --- | --- | --- |
|  |  | Spearman r | 0.624 | | Spearman r | -0.2303 | | Spearman r | 0.2781 | | Spearman r | 0.3685 | |
| All samples |  | 95% CI | 0.2374 to 0.8399 | | 95% CI | -0.6193 to 0.2495 | | 95% CI | -0.2010 to 0.6498 | | 95% CI | -0.1024 to 0.7045 | |
|  |  | *P* (2-tailed) | 0.0033 | | *P* (2-tailed) | 0.3286 | | *P* (2-tailed) | 0.2352 | | *P* (2-tailed) | 0.1099 | |
|  |  | Pairs | 20 | | Pairs | 20 | | Pairs | 20 | | Pairs | 20 | |
| Diagnostic samples | | Spearman r | 0.667 | | Spearman r | -0.4101 | | Spearman r | 0.2731 | | Spearman r | 0.2643 | |
|  |  | 95% CI | 0.1951 to 0.8884 | | 95% CI | -0.7795 to 0.1709 | | 95% CI | -0.3169 to 0.7107 | | 95% CI | -0.3254 to 0.7060 | |
|  |  | *P* (2-tailed) | 0.011 | | *P* (2-tailed) | 0.145 | | *P* (2-tailed) | 0.3420 | | *P* (2-tailed) | 0.3582 | |
|  |  | Pairs | 14 | | Pairs | 14 | | Pairs | 14 | | Pairs | 14 | |
| Sample ID | ITD-AR | Mean Cq | Rel. quantity | SEM | Mean Cq | Rel. quantity | SEM | Mean Cq | Rel. quantity | SEM | Mean Cq | Rel. quantity | SEM |
| AML_059_1* | 0.88 | 23.88 | 3.40 | 1.21 | 30.05 | 0.70 | 0.05 | 23.77 | 1.43 | 0.08 | 22.24 | 4175.84 | 29.33 |
| AML_038_1* | 0.44 | 24.91 | 1.67 | 0.33 | 32.06 | 0.17 | 0.02 | 25.30 | 0.49 | 0.01 | 32.39 | 3.67 | 1.83 |
| AML_060_1* | 0.39 | 24.52 | 2.19 | 0.17 | 28.02 | 2.85 | 0.09 | 28.43 | 0.06 | 0.01 | 28.64 | 49.46 | 3.74 |
| AML_014_1 | 0.39 | 24.89 | 1.69 | 0.58 | 32.19 | 0.16 | 0.00 | 26.43 | 0.23 | 0.01 | 32.98 | 2.43 | 0.86 |
| AML_013_1 | 0.37 | 24.96 | 1.62 | 0.41 | 30.17 | 0.64 | 0.02 | 24.05 | 1.18 | 0.06 | 33.02 | 2.37 | 0.33 |
| AML_024_1 | 0.35 | 25.20 | 1.36 | 0.44 | 31.42 | 0.27 | 0.02 | 25.10 | 0.57 | 0.01 | 33.44 | 1.77 | 0.42 |
| AML_037_1 | 0.34 | 25.23 | 1.33 | 0.42 | 30.28 | 0.60 | 0.02 | 24.01 | 1.21 | 0.04 | 29.80 | 22.13 | 5.69 |
| AML_048_3* | 0.31 | 24.70 | 1.93 | 0.29 | 27.39 | 4.41 | 0.22 | 23.90 | 1.31 | 0.08 | 29.53 | 26.60 | 3.61 |
| AML_026_1 | 0.30 | 25.16 | 1.40 | 0.36 | 32.00 | 0.18 | 0.02 | 25.38 | 0.47 | 0.05 | 30.90 | 10.34 | 16.57 |
| AML_055_1 | 0.26 | 25.66 | 0.99 | 0.15 | 25.91 | 12.26 | 0.13 | 24.97 | 0.62 | 0.02 | 32.48 | 3.46 | 0.72 |
| AML_073_1 | 0.24 | 26.57 | 0.53 | 0.02 | 29.33 | 1.15 | 0.08 | 24.09 | 1.15 | 0.03 | 32.14 | 4.36 | 0.24 |
| AML_083_2* | 0.24 | 25.60 | 1.04 | 0.27 | 29.43 | 1.08 | 0.13 | 26.12 | 0.28 | 0.01 | 30.53 | 13.37 | 2.32 |
| AML_070_1 | 0.24 | 24.92 | 1.65 | 0.14 | 28.34 | 2.28 | 0.16 | 23.89 | 1.32 | 0.22 | 31.08 | 9.14 | 0.11 |
| AML_080_1 | 0.15 | 26.32 | 0.63 | 0.15 | 29.50 | 1.02 | 0.03 | 26.33 | 0.24 | 0.01 | 36.63 | 0.19 | 0.06 |
| AML_007_1* | 0.10 | 26.68 | 0.49 | 0.07 | 31.58 | 0.24 | 0.03 | 30.75 | 0.01 | 0.00 | 31.49 | 6.85 | 0.03 |
| AML_067_1 | 0.07 | 26.42 | 0.58 | 0.04 | 28.43 | 2.14 | 0.11 | 26.61 | 0.20 | 0.00 | 26.10 | 288.09 | 6.38 |
| AML_027_1 | 0.05 | 25.85 | 0.87 | 0.35 | 29.47 | 1.04 | 0.17 | 25.57 | 0.41 | 0.03 | 33.45 | 1.76 | 0.31 |
| AML_018_1 | 0.04 | 25.48 | 1.13 | 0.18 | 28.34 | 2.28 | 0.06 | 25.21 | 0.53 | 0.01 | 33.47 | 1.74 | 0.23 |
| AML_076_1 | 0.04 | 25.61 | 1.03 | 0.30 | 31.90 | 0.19 | 0.03 | 26.93 | 0.16 | 0.01 | 31.80 | 5.52 | 0.49 |
| AML_016_1 | 0.03 | 25.65 | 1.00 | 0.04 | 29.53 | 1.00 | 0.24 | 24.29 | 1.00 | 0.06 | 34.27 | 1.00 | 0.38 |

Footnotes: CI, confidence interval; Rel., relative; SEM, standard error of mean, *, relapsed or refractory AML.

**Supplementary Table 8. Association of *HLF* expression with FLT3 inhibitor response.**

|  | *FLT3*-WT | | | | *FLT3*-ITD+ | | | |
| --- | --- | --- | --- | --- | --- | --- | --- | --- |
|  | Pearson r | 95% CI | P value | Number of XY pairs | Pearson r | 95% CI | P value | Number of XY pairs |
| Tandutinib | -0.2511 | -0.5560 to 0.1133 | 0.1731 | 31 | 0.5069 | 0.0831 to 0.7755 | 0.0225 | 20 |
| Lestaurtinib | -0.3242 | -0.6086 to 0.0341 | 0.0752 | 31 | 0.3127 | -0.1507 to 0.6634 | 0.1795 | 20 |
| Quizartinib | -0.2313 | -0.5413 to 0.1340 | 0.2106 | 31 | 0.4118 | -0.0376 to 0.7226 | 0.0712 | 20 |
| Midostaurin | -0.1625 | -0.4992 to 0.2170 | 0.3998 | 29 | 0.5762 | 0.1321 to 0.8276 | 0.0155 | 17 |
| Sorafenib | -0.2396 | -0.5475 to 0.1254 | 0.1942 | 31 | 0.3453 | -0.1147 to 0.6834 | 0.1359 | 20 |
| Ponatinib | -0.2538 | -0.5579 to 0.1105 | 0.1684 | 31 | 0.3679 | -0.0892 to 0.6969 | 0.1106 | 20 |
| Crenolanib | -0.0973 | -0.4899 to 0.3280 | 0.6587 | 23 | 0.6194 | 0.1038 to 0.8726 | 0.0240 | 13 |
| Linifanib | -0.117 | -0.4758 to 0.2753 | 0.5612 | 27 | 0.5845 | 0.1251 to 0.8376 | 0.0174 | 16 |
| Cabozantinib | -0.1168 | -0.4757 to 0.2754 | 0.5617 | 27 | 0.5245 | 0.0389 to 0.8097 | 0.0370 | 16 |
| Sunitinib | -0.3763 | -0.6447 to -0.0253 | 0.0369 | 31 | 0.4494 | 0.0085 to 0.7440 | 0.0468 | 20 |

**Supplemental Table 9. Clinical responses of four *FLT3*-ITD^+^ AML patients treated with sorafenib.** Therapy responses were evaluated on the basis of ELN 2017 guidelines (8).

| Patient ID | Karyotype | Disease state at therapy start | Treatment | Treatment period (days) | Time to progression (weeks) | Treatment related toxicity | Treatment response | ITD-AR | *HLF* expression (log2CPM) |
| --- | --- | --- | --- | --- | --- | --- | --- | --- | --- |
| **AML_070** | t(3;3)(q21;q26.2), monosomy 7 | Relapse | Sorafenib | 3 + 1 | N/A | N/A | RD | 0.240 | 1.065 |
| **AML_082** | t(6;9)(p23;q34)/  *DEK-NUP214* | R/R | Sorafenib-Clofarabine | 63 | Lost to follow-up | BM  hypocellularity  (gr 4) | Morphologic leukemia-free state | 0.417 | 0.250 |
| **AML_048** | CN-AML | R/R | Azacytidine - Sorafenib - Lenalidomide-Dexamethasone | 64 | 9 | Neutropenic infection (gr3) | PR | 0.481 | 2.730 |
| **AML_059** | t(6;9)(p23;q34)/  *DEK-NUP214* | R/R | Sorafenib | 78 | 7 | Neutropenic infection (gr3) | CRi | 0.879 | 4.378 |

Footnotes: BM, bone marrow; CN, cytogenetically normal; Dg, diagnosis; CRi, complete remission with incomplete hematologic recovery; MRD, minimal residual disease; N/A, not available; PR, partial response; RD, refractory disease; R/R, relapsed/refractory disease.
